# Comparison of clinical characteristics in acute and early HIV-1 infection across different subtypes: a hospital-based cohort study

**DOI:** 10.3389/fmicb.2026.1878265

**Published:** 2026-07-17

**Authors:** Shanshan Tang, Tianyang Liu, Zifan Zhang, Tingdan Gong, Yao Wang, Xiaowen Li, Qiuyue Zhang, Fuxiang Wang, Hongzhou Lu, Lukun Zhang, Yun He, Lanlan Wei

**Affiliations:** 1 People’s Hospital of Naxi District, Luzhou, Sichuan, China; 2 Department of Infectious Diseases, The Affiliated Hospital of Southwest Medical University, Luzhou, Sichuan, China; 3 Institute for Hepatology, National Clinical Research Center for Infectious Diseases, Shenzhen Third People’s Hospital, Shenzhen, Guangdong, China; 4 Department of Microbiology, School of Basic Medical Sciences, Harbin Medical University, Heilongjiang Provincial Key Laboratory of Infection and Immunity, Harbin, China; 5 Department of Infectious Diseases, Shenzhen Third People’s Hospital, Shenzhen, Guangdong, China

**Keywords:** acute early HIV infection, antiretroviral therapy, HIV-1 subtype, molecular epidemiology, transmitted drug resistance, viral suppression

## Abstract

**Background:**

Early diagnosis and immediate antiretroviral therapy (ART) for acute and early HIV-1 infection (AEHI) effectively curb HIV transmission and restrict viral reservoir formation. However, subtype-specific transmitted drug resistance (TDR) and early ART virological response remain insufficiently characterized among circulating HIV-1 recombinants in the Guangdong-Hong Kong-Macao Greater Bay Area. This study aimed to compare these key indicators across different HIV-1 subtypes.

**Methods:**

This hospital-based cohort study enrolled 105 AEHI patients from 2019 to 2024. HIV-1 pol gene sequencing was performed for subtype classification and drug resistance genotyping. Antiretroviral therapy responses were monitored at weeks 4, 12, 24, and 48. Demographic, clinical, virological, immunological, and TDR data were systematically analyzed across all HIV-1 subtypes.

**Results:**

CRF07_BC was the dominant strain (32.4%), followed by other BC recombinants (30.5%) and CRF01_AE (22.9%). Poppers use was significantly associated with CRF07_BC transmission among MSM (*P* = 0.007). The B+CRF01_AE/CRF55_01B group exhibited an extremely high TDR rate of 90.9% (*P* < 0.001), dominated by V179E and M184V mutations. At weeks 12 and 24 post-ART, BC recombinants and B+CRF01_AE/CRF55_01B showed slower viral suppression than CRF07_BC, while all subtypes achieved comparable long-term immune recovery (92.6% virological suppression at week 48).

**Conclusion:**

Significant heterogeneity in TDR and early viral load suppression exists among HIV-1 subtypes during acute infection. Baseline drug resistance testing and intensified viral load monitoring are recommended for patients with B/CRF01_AE recombinant strains. These findings support regional HIV molecular surveillance and subtype-targeted early intervention.

## 1 Introduction

Diagnosing and treating acute HIV infection (AHI) and early HIV infection (EHI) are crucial for improving patient outcomes and reducing transmission. AHI represents the early stage of HIV infection in the human body, during which individuals may experience acute retroviral syndrome (ARS). The symptoms of ARS are nonspecific and often misdiagnosed as other viral infections (Cowan et al., 2021). In this stage, the viral load in individuals is relatively high, making them 26 times more infectious than during the asymptomatic period (Hollingsworth et al., 2008). However, AHI and EHI are frequently not diagnosed promptly, with late-stage HIV diagnosis rates in China ranging from 29.78% to 52.63%, indicating that many individuals are diagnosed only in advanced stages of the infection (Chu et al., 2023; Sheehan et al., 2017; Wilton et al., 2019). Early identification of AHI or EHI and the initiation of Antiretroviral Therapy (ART) can reduce immune system damage and decrease viral reservoirs (Leyre et al., 2020).

The global genetic diversity of HIV-1 strains is a notable characteristic of the virus (Jiamsakul et al., 2016). In China, the vast geographic range and high population mobility contribute to the continuous emergence of novel recombinant HIV viruses that spread rapidly among the population (Ge et al., 2022). Research by Academician Hong Shang and colleagues revealed that among men who have sex with men (MSM) in northeastern China, all individuals newly infected with the HIV-1 CRF01_AE virus were infected with R5-type viruses, which target the CCR5 coreceptor on helper T cells (Cui et al., 2019). The early transition from R5 to X4/DM types is linked to a rapid decline in CD4+T cell counts (Ge et al., 2021a; Marichannegowda et al., 2023). A case report noted a significant increase in the virulence of a specific HIV-1 recombinant strain, which accelerated the depletion of CD4+T cell counts and led to a markedly faster progression from acute infection to AIDS, despite no mutations in drug-resistant sites (Markowitz et al., 2005; Zhang J. et al., 2021). However, comprehensive studies on clinical, virological, and immunological differences among various strains are limited. Due to the rapid mutation of HIV strains and significant regional differences, research on the clinical characteristics of different subtypes during the acute/early infection period in the Shenzhen region is lacking.

There is scant research on the correlation between clinical characteristics of acute HIV infection and different subtypes, both domestically and internationally. Most studies have concentrated on the relationship between ARS and inflammatory factors following AHI or EHI, with little exploration of clinical characteristics and differences in transmission drug resistance among subtypes during the early stages of infection (Crowell et al., 2018; De Clercq et al., 2024; Hassan et al., 2021). Thus, it is imperative to investigate the clinical characteristics of acute HIV infection across different subtypes.

Conducting research on the clinical characteristics of early infection with different HIV subtypes is essential to differentiate the acute infection profiles of these subtypes. This research will provide a foundation for diagnosis and treatment evaluation in clinical practice.

## 2 Materials and methods

### 2.1 Study design

**FIGURE 1:**
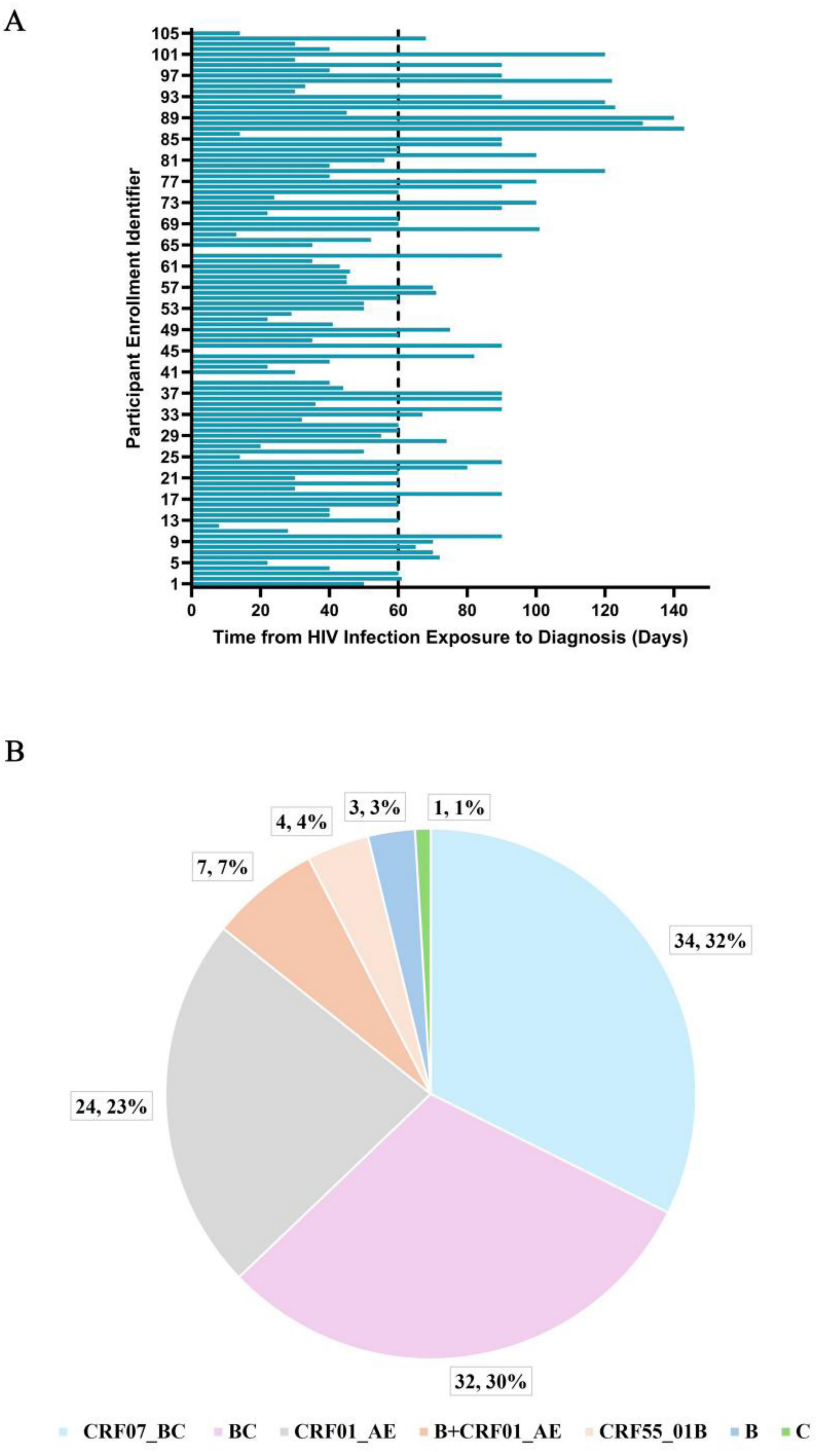
Overall information about the participants. **(A)** The number of days from suspected exposure to the confirmed diagnosis of AHI and EHI for each enrolled participant. The dotted vertical line represents the median time (60 days) from suspected HIV exposure to confirmed diagnosis; **(B)** The subtype distribution of the infecting strains.

This cohort study of adult acute HIV infections was conducted at a Shenzhen hospital from 2019 to 2024. Participants diagnosed with acute or early HIV infection by experienced physicians were enrolled and followed for 1 year after initiating antiretroviral therapy. Epidemiological history, symptom assessment, and physical examination were performed. Collected data included high-risk exposures within 6 months, condom use during sexual activity, HIV antibody test results (hospital or self-reported), confirmatory test outcomes, and fourth-generation ELISA antigen/antibody detection. The interval between the most recent HIV-negative test (antigen/antibody, confirmatory, or RNA) and the reported exposure determined the exposure-to-diagnosis duration, which was staged according to Fiebig: Stage I (≤ 5 days), II (6–10 days), III (11–14 days), IV (15–20 days), V (21–89 days), and VI (90–180 days) (Fiebig et al., 2003). The median time from suspected exposure risk to the diagnosis of HIV infection for most of the enrolled patients was 60[8-143] days (Figure 1A). Upon diagnosis, participants were initiated on ART based on their clinical condition. Follow-up visits were scheduled at the following time points: 2 weeks (2W), 4 weeks (4W), 8 weeks (8W), 12 weeks (12W), 24 weeks (24W), 36 weeks (36W), and 48 weeks (48W), in addition to the baseline (0 weeks). During the study, two participants were lost to follow-up, and only partial baseline data were recorded for them, a total of 103 participants had follow-up information.

### 2.2 Study population and sampling strategy

Participants were included if they were aged 20–65 years, of any gender, and diagnosed with AHI and EHI at a hospital in Shenzhen. They needed to have a history of high-risk exposure to HIV within the past 6 months and exhibit detectable virus in plasma (p24 antigen and/or HIV RNA) and/or negative or indeterminate HIV antibodies, regardless of whether they had clinical symptoms (Cowan et al., 2021; Gulick et al., 2024; Rockstroh et al., 2023). The primary exclusion criteria were patients with a documented or self-reported positive HIV antibody test and a confirmed HIV-1 diagnosis (via Western blot) for more than 6 months, or those who could not clearly report the time of high-risk exposure and had a negative HIV antibody test within the past 6 months. Other exclusion criteria included patients who were confirmed HIV-negative within the past 6 months, those who were pregnant or breastfeeding, or individuals with mental health disorders or other conditions that could interfere with participation in the study. Our study employed a random sampling strategy, selecting participants from individuals diagnosed with early HIV infection between 2019 and 2024.

### 2.3 Data collection

Trained health personnel conducted face-to-face interviews with the participants. The socio-demographic characteristics assessed included age, gender, education level, monthly income, marital status, smoking habits, alcohol consumption, substance use, and physical exercise. Inquiries also covered behavioral traits such as sexual orientation, exposure history, tobacco and alcohol use, substance utilization, and exercise routines. Additionally, data were collected regarding the history of sexually transmitted infections, including hepatitis B and syphilis. Baseline indicators for individuals with AHI and EHI were gathered, including duration of infection, viral load, total HIV DNA, transmitted drug resistance mutation, CD4+ T cell counts, CD8+T cell counts, lymphocyte levels, neutrophil counts, monocyte counts, platelet counts, hemoglobin levels, transaminase values, creatinine levels, glomerular filtration rate, cholesterol levels, triglyceride levels, and thyroid function.

### 2.4 HIV typing detection

After the subjects were enrolled and signed the informed consent form, 10 mL of whole blood was collected from each participant and transported to the Dongguan Hailit Laboratory within 6 h using ice packs. Total viral RNA was then extracted from the plasma, separated from the blood samples, using a high-purity viral RNA extraction kit. Subsequently, nested reverse transcription polymerase chain reaction (RT-PCR) was performed with a one-step RNA PCR and ExTaq kit. Multiple primers and specific thermal cycling conditions were used to amplify the protease and reverse transcriptase fragments of the pol gene, as well as the integrase gene fragment. The samples were then sent to an external laboratory for sequencing. All sequencing data were submitted to the HIV-1 sequence quality control tool^1^ for sequence quality verification. All sequence alignments were performed against the standard HIV-1 HXB2 reference genome (GenBank accession number: K03455). The protease and reverse transcriptase gene sequencing data derived from the pol gene of each participant will be fully deposited in GenBank before the official online publication of this manuscript, and complete GenBank accession numbers will be supplemented in the final version upon availability. The LANL HIV Database only provides analytical pipelines including HIV BLAST, RIP, and jpHMM, which we utilized to screen and characterize recombinant HIV strains from our aligned sequences. Phylogenetic trees were constructed in MEGA 11 software using the Kimura two-parameter model, with 1,000 bootstrap replicates to determine the subtype of the sequences. All curated pol gene sequences were imported into the Stanford HIV Drug Resistance Database for systematic identification and annotation of transmitted drug resistance mutation.

### 2.5 Statistical analysis

All statistical analyses were conducted using SPSS version 26, GraphPad Prism version 9, and R version 4.4.0, with statistical significance set at *P* < 0.05. Descriptive statistics were used to summarize baseline characteristics and clinical parameters of the patients. The Kruskal-Wallis’s test was employed to compare the mean laboratory results across multiple independent samples and to examine differences in follow-up data among various subtypes. Chi-square tests and Fisher’s exact tests were applied to assess associations between categorical variables. The study endpoint was defined as viral load (HIV-1 RNA) ≥200 copies/mL at different time points, and Kaplan-Meier curves were utilized to estimate the probability of maintaining a viral load ≥200 copies/mL after ART initiation across subtypes. LOESS (Locally Weighted Regression) analysis was conducted to depict the dynamic changes in CD4+ T-cell counts and CD4+/CD8+ T-cell ratios during follow-up.

## 3 Results

### 3.1 Suspected exposure duration in acute and early HIV-1 infection

This study was conducted at the only designated infectious disease hospital in Shenzhen, a core metropolis in the Guangdong-Hong Kong-Macao Greater Bay Area featured by high cross-border population mobility and active HIV-1 recombinant evolution. A total of 105 individuals with acute and early HIV-1 infection were analyzed, revealing a median self-reported interval of 60 days from suspected exposure to diagnosis confirmation (IQR 37–89 days, range: 8–143 days), with the complete distribution depicted in Figure 1A. Notably, three participants (2.86%, 3/105) could not recall a specific exposure date; however, epidemiological tracing confirmed their exposure-to-diagnosis intervals did not exceed 180 days, thus they were included in the early-infection cohort. Regarding Fiebig staging, 95 individuals (90.47%, 95/105) sought medical care during stages V–VI of acute HIV infection (corresponding to approximately 21–180 days post-infection), indicating this period serves as the primary window for early clinical diagnosis. In contrast, seven patients (6.67%, 7/105) were diagnosed at stages II–IV (6–20 days post-infection). This subgroup exhibited a distinct demographic profile: all seven were MSM aged under 40 years (range: 22–38 years).

### 3.2 Subtype distribution in acute and early HIV-1 infection

Sanger sequencing and sequence alignment were performed on plasma samples from 105 individuals with acute and early HIV-1 infection, and the subtype assignment was carried out based on protease (PR) and reverse transcriptase (RT) gene sequences. The circulating HIV-1 subtypes were identified as follows: CRF07_BC (32.4%, 34/105), other BC recombinants (30.5%, 32/105), CRF01_AE (22.9%, 24/105), B + CRF01_AE (6.7%, 7/105), CRF55_01B (3.8%, 4/105), subtype B (2.9%, 3/105), and subtype C (1.0%, 1/105) (Figure 1B). Because CRF55_01B represents a recombinant form of CRF01_AE and subtype B (Ge et al., 2022; Han et al., 2013), and phylogenetic analysis showed that CRF55_01B and B + CRF01_AE clustered together (Supplementary Figure 1), these two subtypes were grouped as a single category for subsequent comparisons with other subtypes. Within this early-infection cohort, pure subtypes B and C comprised only a small fraction of cases; consequently, they were excluded from the subtype-stratified analyses of baseline characteristics and follow-up outcomes. Finally, 101 participants were included for all baseline statistical analyses.

### 3.3 Acute and early HIV-1 infection demographics by subtype

The demographic characteristics of participants with AHI and EHI showed no significant differences across subtypes, except for differences in rush poppers use and physical exercise (Table 1). Regarding the use of rush poppers, participants infected with the CRF07_BC subtype (44.1%, 15/34) had a significantly higher rate of rush poppers use compared to other subtypes (*P* = 0.007). In terms of physical exercise, participants infected with the CRF07_BC subtype (26.5%, 9/34) were significantly less likely to engage in regular physical exercise compared to participants with other subtypes (*P* = 0.044). The majority of AHI and EHI participants were male (98.0%, 99/101), with most aged between 20 and 49 years (95.0%, 96/101), and a small proportion aged 50 years or older (5.0%, 5/101). Most participants in the cohort were MSM (88.1%, 89/101), although a minority were bisexual (5.0%, 5/101). However, all bisexual participants reported suspected exposure risk exclusively through male-to-male sexual contact. Educational attainment was generally high among the cohort, with 74.7% (74/99) having completed university or college-level education. Most participants were unmarried (83.0%, 83/101). In terms of monthly income, the majority of participants in the CRF07_BC group (60.0%, 18/30) reported a monthly income exceeding 10,000 RMB, whereas only a minority of participants in the BC group (34.8%,8/23), CRF01_AE group (35.0%,7/20), and B+CRF01_AE/CRF55_01B group (28.6%, 2/7) reported the same. However, no significant differences were observed across subtypes (*P* = 0.184). Regarding lifestyle habits, few participants reported smoking (17.6%, 16/91) or drinking alcohol (7.7%, 7/91).

**TABLE 1:** Demographic characteristics of AHI and EHI cases among participants with different HIV-1 subtypes.

Characteristic	CRF07_BC	BC	CRF01_AE	B+CRF01_AE/CRF55_01B	χ^2^	*P*-value
Age group (y)					11.058	0.212
18∼29	18 (52.9)	15 (46.9)	11 (45.8)	9 (81.8)		
30∼39	12 (35.3)	10 (31.3)	10 (41.7)	0 (0)		
40∼49	3 (8.8)	5 (15.6)	1 (4.2)	2 (18.2)		
≥ 50	1 (2.9)	2 (6.3)	2 (8.3)	0 (0)		
Sex					2.005	0.785
Male	33 (97.1)	32 (100.0)	23 (95.8)	11 (100.0)		
Female	1 (2.9)	0 (0)	1 (4.2)	0 (0)		
Sexual orientation					2.137	0.974
Homosexuality	29 (85.3)	29 (90.6)	21 (87.5)	10 (90.9)		
Heterosexuality	2 (5.9)	2 (6.3)	2 (8.3)	1 (9.1)		
Bisexuality	3 (8.8)	1 (3.1)	1 (4.2)	0 (0)		
Exposure history					3.409	0.862
MSM	32 (94.2)	30 (93.8)	22 (91.7)	10 (90.9)		
Heterosexual male	1 (2.9)	2 (6.3)	1 (4.2)	1 (9.1)		
Heterosexual female	1 (2.9)	0 (0)	1 (4.2)	0 (0)		
Education level^a^					9.994	0.256
Primary school or lower	1 (2.9)	0 (0)	2 (8.7)	0 (0)		
Junior high school	4 (11.8)	4 (12.9)	0 (0)	0 (0)		
Senior high school	3 (8.8)	3 (9.7)	5 (21.7)	3 (27.3)		
College degree or higher	26 (76.5)	24 (77.4)	16 (69.6)	8 (72.7)		
Marital status at enrollment					2.042	0.948
Single	27 (79.4)	26 (81.3)	20 (83.4)	10 (90.9)		
Married	5 (14.7)	5 (15.6)	2 (8.3)	1 (9.1)		
Divorced	2 (5.9)	1 (3.1)	2 (8.3)	0 (0)		
Monthly income^b^					10.816	0.184
<2500RMB	1 (3.3)	0 (0)	1 (5.0)	0 (0)		
2500∼5000RMB	2 (6.7)	1 (4.3)	0 (0)	1 (14.3)		
5000-10000RMB	9 (30.0)	14 (60.9)	12 (60.0)	4 (57.1)		
>10000RMB	18 (60.0)	8 (34.8)	7 (35.0)	2 (28.6)		
Smoking^a^					1.823	0.630
Yes	6 (17.6)	5 (18.5)	5 (22.7)	0 (0)		
No	28 (82.4)	22 (81.5)	17 (77.3)	8 (100.0)		
Drinking alcohol^c^					2.181	0.507
Yes	2 (5.9)	4 (14.8)	1 (4.5)	0 (0)		
No	32 (94.1)	23 (85.2)	21 (95.5)	8 (100.0)		
Using rush poppers^c^					**11.442**	0.007**
Yes	15 (44.1)	2 (7.4)	7 (31.8)	1 (12.5)		
No	19 (55.9)	25 (92.6)	15 (68.2)	7 (87.5)		
Physical exercise^d^					**7.984**	0.044*
Yes	9 (26.5)	16 (61.5)	11 (50.0)	4 (50.0)		
No	25 (73.5)	10 (38.5)	11 (50.0)	4 (50.0)		

N, total number of participants included in baseline comparative analyses = 101; n, number of individuals within each HIV-1 subtype subgroup. All categorical variables are expressed as *n* (%).

^a^The BC group has one individual with missing data, and the CRF_01AE group also has one individual with missing data.

^b^The CRF01_AE group has one individual with missing data, the CRF07_BC group has four individuals with missing data, the BC group has nine individuals with missing data, and the B+CRF01_AE/CRF55_01B group has four individuals with missing data.

^c^The CRF01_AE group has two individuals with missing data, the BC group has five individuals with missing data, and the B+CRF01_AE/CRF55_01B group has three individuals with missing data.

^d^The CRF01_AE group has two individuals with missing data, the BC group has six individuals with missing data, and the B+CRF01_AE/CRF55_01B group has three individuals with missing data.

Bold values represent statistically significant inter-subtype differences, *P < 0.05, **P < 0.01, ***P < 0.001.

### 3.4 Baseline characteristics of acute and early HIV-1 infections by subtype

Analysis of ARS and baseline clinical characteristics by HIV-1 subtype is summarized in Table 2. Overall, ARS incidence failed to differ significantly across subtypes (χ^2^ = 2.257, *P* = 0.523). Similarly, the median interval from suspected exposure to ARS onset also showed no significant differences (χ^2^ = 3.286, *P* = 0.350). Fisher’s exact test, which evaluated the proportion of participants with intervals exceeding 19 days, likewise revealed no subtype-specific differences (*P* = 0.451). However, the BC (66.7%, 8/12) and B+CRF01_AE/CRF55_01B (60.0%, 3/5) groups exhibited higher proportions above this threshold (Supplementary Figure 2). Collectively, among early HIV-1 infections, subtype was not associated with statistically significant differences in ARS incidence, the exposure-to-ARS interval, or baseline Fiebig stage (all *P* > 0.05).

**TABLE 2:** Baseline clinical, virological, and immunological characteristics of AHI and EHI cases among participants with different HIV-1 subtypes.

Clinical characteristics	CRF07_BC	BC	CRF01_AE	B+CRF01_AE/ CRF55_01B	χ^2^/H	*P*-value
ARS^a^					2.257	0.523
Yes	23 (67.6)	18 (56.3)	18 (75.0)	7 (63.6)	3.286	0.350
No	11 (32.4)	14 (43.8)	6 (25.0)	4 (36.4)		
The time from exposure risk to the onset of ARS (days)[Median (IQR)]	19 (7∼110)	19.5 (7∼30)	14 (4∼50)	27 (10∼60)		
Fiebig staging^b^					4.032	0.864
I∼II	1 (2.9)	0 (0)	0 (0)	0 (0)		
III∼IV	1 (2.9)	1 (3.3)	2 (8.3)	0 (0)		
V∼VI	32 (94.1)	29 (96.7)	22 (91.7)	10 (100.0)		
Virological parameters						
Plasma viral load (log_10_ copies/mL) [Median (IQR)]	5.53 (3.92∼9.10)	5.72 (3.75∼8.08)	5.74 (3.05∼7.25)	6.21 (3.61∼7.15)	2.283	0.516
Total HIV DNA (copies/10^6 CD4 T cells)^c^ [Median (IQR)]	163.66	2110.00 (53.42∼9900.00)	341.88 (109.96∼408.11)	1130.00 (60.76∼132000.00)	2.571	0.277
Transmitted drug resistance mutation					**25.995**	<0.001***
Yes	9 (26.5)	3 (9.4)	4 (16.7)	10 (90.9)		
No	25 (73.5)	29 (90.6)	20 (83.3)	1 (9.1)		
Immune cell count						
CD4+T cells (/μL) [Median (IQR)]	343 (78∼838)	297 (137∼556)	347 (147∼838)	348 (192∼704)	1.280	0.734
CD8+T cells (/μL) [Median (IQR)]	1252 (425∼5457)	1168 (395∼3092)	1196 (479∼4130)	1419 (395∼4615)	0.478	0.924
CD4+T cells/CD8+T cells ratio [Median (IQR)]	0.31 (0.03∼0.75)	0.28 (0.07∼1.14)	0.31 (0.05∼1.26)	0.20 (0.11∼0.62)	0.502	0.919
Monocytes (10^3/μL) [Median (IQR)]	0.49 (0.28∼1.18)	0.53 (0.35∼0.86)	0.53 (0.23∼1.20)	0.63 (0.47∼0.76)	2.970	0.396
Neutrophils (10^3/μL) [Median (IQR)]	3.04 (1.10∼6.24)	2.96 (1.23∼4.34)	2.87 (1.54∼5.65)	3.22 (2.17∼7.62)	3.144	0.370
Lymphocyte (10^3/μL) [Median (IQR)]	2.19 (1.20∼5.68)	2.00 (1.34∼6.02)	2.19 (1.09∼3.98)	2.37 (0.83∼5.51)	0.518	0.915

N, total number of participants included in baseline comparative analyses = 101; n, number of individuals within each HIV-1 subtype subgroup. Categorical variables are presented as n (%). All continuous variables are reported as median (interquartile range, IQR), with the statistical label (Median (IQR)) marked separately after each corresponding variable name in the table.

^a^The presence of two or more symptoms, combined with neurological symptoms, liver dysfunction, reduced platelet count, and cholecystitis, is considered indicative of ARS.

^b^The BC group has two individuals with missing data, while the B+CRF01_AE/CRF55_01B group has one individual with missing data. However, the suspected exposure time for these three participants was within 6 months of their diagnosis. Among these 101 individuals, only 19 underwent total HIV DNA testing. Specifically, this included one individual from the CRF07_BC group, ten from the BC group, three from the CRF01_AE group, and five from the B+CRF01_AE/CRF55_01B group.

Bold values represent statistically significant inter-subtype differences, *P < 0.05, **P < 0.01, ***P < 0.001.

Comparison of baseline testing results across subtypes showed that the B+CRF01_AE/CRF55_01B subtype exhibited a higher rate of transmitted drug resistance (90.9%, 10/11) compared to the CRF07_BC subtype (26.5%, 9/34), BC subtype (9.4%, 3/32), and CRF01_AE subtype (16.7%, 4/24) (*P* < 0.001) (Table 2). The transmitted drug resistance mutations identified in the B+CRF01_AE/CRF55_01B group included non-nucleoside reverse transcriptase inhibitor (NNRTI)-related V179E (81.8%, 9/11), nucleoside reverse transcriptase inhibitor (NRTI)-related M184V (18.2%, 2/11), NNRTI-related Y181C (9.1%, 1/11), NNRTI-related E138G (9.1%, 1/11), and NNRTI-related V179VDE (9.1%, 1/11). In the CRF07_BC group, transmitted drug resistance mutations included NNRTI-related V179D (11.8%, 4/34), protease (PR)-related K43T (5.9%, 2/34), NRTI-related S68G (2.9%, 1/34), NNRTI-related E138EK (2.9%, 1/34), and integrase (IN)-related E157EQ (2.9%, 1/34). In the BC group, transmitted drug resistance mutations included NNRTI-related V179D (6.3%, 2/32), NRTI-related M184V (6.3%, 2/32), and NNRTI-related K103N (3.1%, 1/32). In the CRF01_AE group, transmitted drug resistance mutations included NNRTI-related V179E (4.2%, 1/24), K103N (4.2%, 1/24), and N348X (4.2%, 1/24), as well as PR-related L33F (4.2%, 1/24). Although plasma HIV-1 RNA levels (log_10_ copies/mL) did not differ significantly among subtypes (H = 2.283, *P* = 0.516; Table 2), the B+CRF01_AE/CRF55_01B group exhibited a higher median plasma HIV-1 RNA (log_10_ copies/mL) than the other subtypes (Supplementary Figure 3).

Baseline immunological parameters did not differ significantly among acute and early HIV-1 infection subtypes (all *P* > 0.05). Although the B+CRF01_AE/CRF55_01B group had a lower median CD4^+^/CD8^+^ T-cell ratio compared to other subtypes, this was not statistically significant (χ^2^ = 0.502, *P* = 0.919). Fisher’s exact test likewise revealed no significant differences in the distributions of immunological markers, including CD4^+^ T-cell count distribution (Supplementary Figure 4B) (all *P* > 0.05). Notably, however, the B+CRF01_AE/CRF55_01B subtype showed higher proportions of: CD4^+^/CD8^+^ ratios ≤ 0.2 (54.5%, 6/11; Supplementary Figure 4A); elevated monocyte counts (54.5%, 6/11; Supplementary Figure 4C); abnormal lymphocyte counts (27.3%, 3/11; Supplementary Figure 4D).

Additionally, a small number of participants (16.1%, 14/87) exhibited abnormal thyroid function at baseline (Table 3). The baseline levels of triiodothyronine (T3) and free triiodothyronine (FT3) varied across the different subtypes (Table 3). Compared to the CRF07_BC group, participants in the B+CRF01_AE/CRF55_01B group had significantly higher T3 (*P* = 0.0256) and FT3 (*P* = 0.0469) levels. Similarly, compared to the BC group, B+CRF01_AE/CRF55_01B participants had higher T3 (*P* = 0.0394) and FT3 (*P* = 0.0072) levels (Supplementary Figures 5A,B). However, most participants had T3 and FT3 levels within the normal reference range, with only a few showing abnormal T3 levels (4.6%, 4/87) and FT3 levels (3.4%, 3/87). No significant statistical differences were observed across subtypes in other baseline symptoms, signs, and test results. A small proportion of tissue damage–related enzymes, such as AST (18.2%, 2/11) and LDH (40.0%, 4/10), showed higher rates of abnormal elevation in the B+CRF01_AE/CRF55_01B subtype compared to other groups (Supplementary Figures 6A,B).

**TABLE 3:** Baseline laboratory findings of AHI and EHI cases among participants with different HIV-1 subtypes.

Laboratory parameters	CRF07_BC	BC	CRF01_AE	B+CRF01_AE/CRF55_01B	χ^2^/H	*P*-value
With sexually trans-mitted diseases						
Hepatitis B	2 (5.9)	2 (6.3)	0 (0)	0 (0)	1.713	0.651
Syphilis	14 (41.2)	14 (43.8)	8 (33.3)	3 (27.3)	1.286	0.761
Pathogen detection						
CMV-DNA (+)^a^	3 (9.4)	0 (0)	3 (13.0)	2 (20.0)	6.239	0.065
Interferon gamma release assay (+)^b^	1 (3.0)	4 (12.5)	1 (4.3)	0 (0)	2.613	0.405
Biochemical parameters						
AST (U/L) [Median (IQR)]	24.0 (16.0∼311.0)	27.2 (12.0∼127.0)	26.0 (12.0∼179.0)	23.0 (12.0∼41.8)	1.818	0.611
ALT (U/L) [Median (IQR)]	28.0 (10.0∼406.0)	29.5 (9.0∼289.0)	30.0 (9.0∼226.0)	28.0 (10.0∼97.6)	1.120	0.772
LDH(U/L) [Median (IQR)]	208 (127∼614)	213 (161∼811)	212 (130∼374)	216 (117∼389)	2.691	0.442
CK(U/L) [Median (IQR)]	85 (31∼742)	77 (32∼592)	94 (48∼505)	85 (45∼163)	3.297	0.348
ESR (mm/h) [Median (IQR)]	22 (3∼52)	22 (2∼105)	30 (14∼46)	30 (2∼55)	5.828	0.120
Cholesterol (mmol/L) [Median (IQR)]	4.12 (3.02∼5.64)	4.02 (2.79∼6.77)	4.05 (2.48∼6.55)	4.09 (3.08∼5.74)	0.038	0.998
Triglyceride (mmol/L) [Median (IQR)]	1.48 (0.62∼3.31)	1.50 (0.51∼9.37)	1.46 (0.53∼3.75)	0.96 (0.66∼2.70)	3.139	0.371
Creatinine (umol/L) [Median (IQR)]	77.0 (50.7∼131.0)	79.5 (63.8∼118.0)	76.5 (62.0∼118.0)	73.0 (62.0∼112.0)	1.797	0.616
GFR (ml/min) [Median (IQR)]	112.7 (62.1∼135.7)	110.9 (62.1∼129.8)	111.7 (55.1∼128.9)	111.0 (79.9∼129.7)	0.623	0.891
TSH (mIU/L) [Median (IQR)]	1.75 (0.21∼4.56)	1.92 (0.70∼3.31)	1.73 (0.47∼3.30)	1.92 (1.20∼4.99)	2.577	0.462
T3 (nmol/L) [Median (IQR)]	1.88 (1.07∼2.72)	1.90 (1.09∼2.95)	2.11 (1.16∼2.66)	2.04 (1.67∼3.18)	**14.149**	0.003**
T4 (nmol/L) [Median (IQR)]	105.5 (73.0∼148.0)	115.5 (74.0∼165.0)	115.5 (63.0∼229.0)	123.0 (86.1∼165.0)	7.770	0.051
FT3 (pmol/L) [Median (IQR)]	5.27 (4.04∼7.61)	5.23 (2.67∼6.75)	5.38 (3.89∼7.37)	5.88 (5.38∼6.85)	**11.635**	0.009**
FT4 (pmol/L) [Median (IQR)]	18.3 (14.7∼25.5)	18.4 (12.2∼23.8)	18.4 (13.7∼29.1)	18.4 (16.3∼25.9)	1.217	0.749

N, total number of participants included in baseline comparative analyses = 101; n, number of individuals within each HIV-1 subtype subgroup. Categorical variables are presented as *n* (%). All continuous variables are reported as median (interquartile range, IQR), with the statistical label (Median (IQR)) marked separately after each corresponding variable name in the table.

^a^The CRF_07BC group has two individuals with missing data; the CRF_01AE group has one individual with missing data; and the B+CRF01_AE/CRF55_01B group has one individual with missing data.

^b^The CRF_07BC group has one individual with missing data, and the CRF_01AE group also has one individual with missing data. ARS, acute retroviral syndrome; CMV, Cytomegalovirus; AST, aspartate aminotransferase; ALT, alanine aminotransferase; LDH, lactate dehydrogenase; CK, creatine kinase; ESR, erythrocyte sedimentation rate; GFR, glomerular filtration rate; TSH, thyroid - stimulating hormone; T3, triiodothyronine; T4, thyroxine; FT3, free triiodothyronine; FT4, free thyroxine.

Bold values represent statistically significant inter-subtype differences, *P < 0.05, **P < 0.01, ***P < 0.001.

### 3.5 Post-ART follow-up dynamics in acute and early HIV-1 infections by subtype

In the cohort study, two participants were lost to follow-up during treatment preparation, and another two began ART more than 30 days after diagnosis. Besides these four cases, four more participants were excluded at baseline, leaving 97 participants for final analyses, 81 participants provided valid viral load measurements at week 48. The remaining participants all started ART within 30 days of diagnosis. The majority (76.3%, 74/97) of those initiating treatment received the bictegravir/emtricitabine/tenofovir alafenamide (BIC/FTC/TAF) regimen. During the 48-week follow-up, three participants who started with BIC/FTC/TAF switched to alternative regimens: two due to renal dysfunction, switching to dolutegravir/lamivudine, and one switched to zidovudine/lamivudine/efavirenz (AZT/3TC/EFV) following a tuberculosis diagnosis to avoid potential drug interactions. A small number of participants (10.3%, 10/97) with lower viral loads and poorer economic conditions started treatment with tenofovir/lamivudine/efavirenz (TDF/3TC/EFV) as their initial regimen. However, one participant switched to BIC/FTC/TAF due to hepatitis C infection to avoid drug interactions. A very small group of participants (6.2%, 6/97) with baseline renal impairment and low viral load initiated treatment with dolutegravir/lamivudine (DTG/3TC), and no changes were made to their regimen during the 48-week follow-up. Another small group (6.2%, 6/97) started with lopinavir/ritonavir/lamivudine/tenofovir (LPV/r/3TC/TDF), with one participant switching to rilpivirine/lamivudine/tenofovir (RPV/3TC/TDF) due to diarrhea, and two others switched to BIC/FTC/TAF due to poor treatment response. Only one participant began treatment with ainuovirine/lamivudine/tenofovir (ANV/3TC/TDF), but due to poor treatment response, they switched to the BIC/FTC/TAF regimen.

**FIGURE 2:**
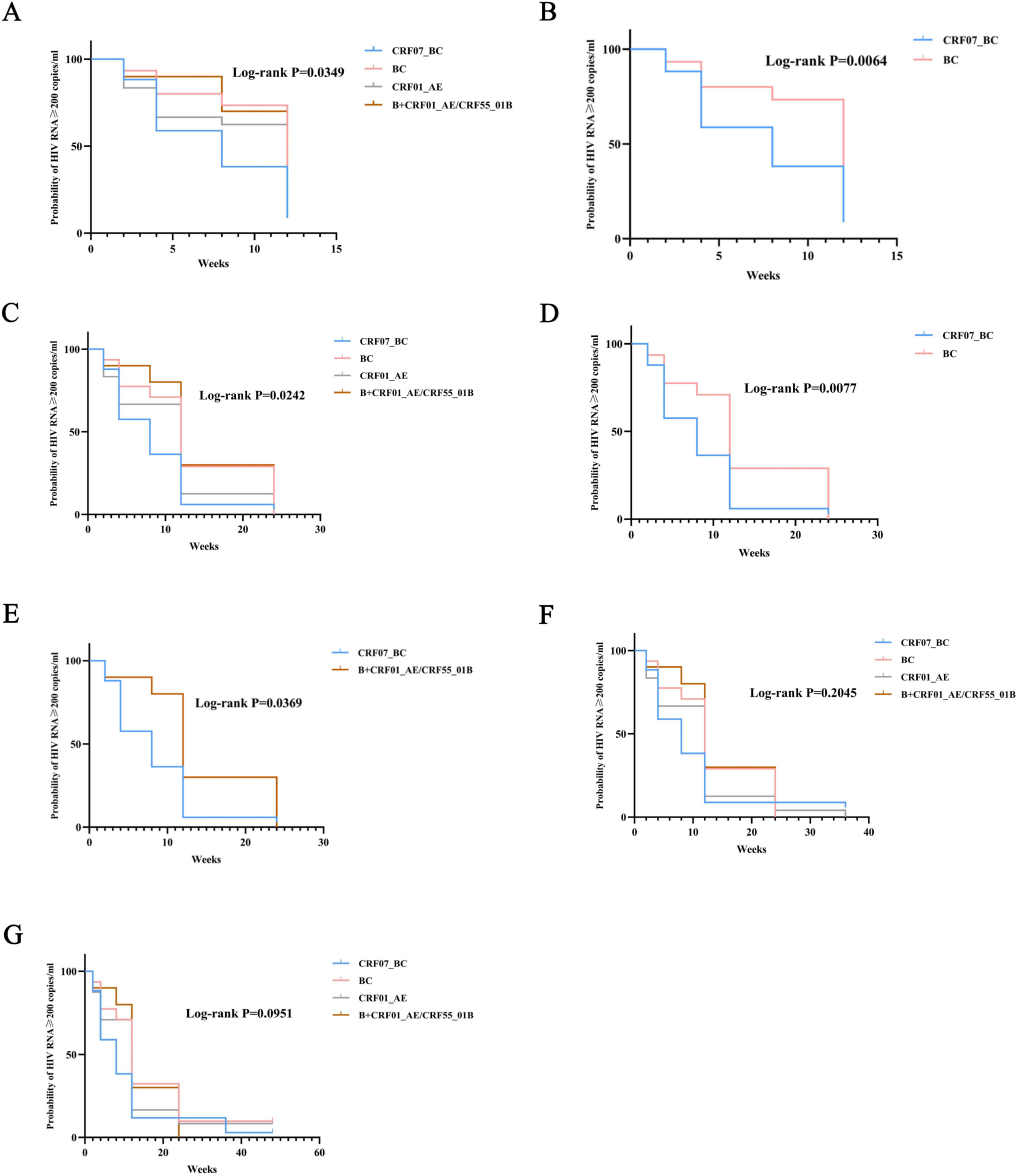
Kaplan–Meier curves showing the proportion of early HIV-1–infected individuals with HIV-RNA ≥ 200 copies/mL at successive intervals following ART initiation. **(A)** and **(B)** display data through 12 weeks; **(C–E)** extend to 24 weeks; **(F)** covers 36 weeks; and **(G)** covers 48 weeks. Each curve represents a different subtype group (CRF07_BC; other BC recombinants; CRF01_AE; and the combined B + CRF01_AE/CRF55_01B category). *P*-values from Log-rank tests—shown within each panel—indicate statistically significant differences between subtypes.

Among acute and early HIV-1–infected individuals initiating antiretroviral therapy (ART), viral load dynamics were analyzed over time. At 12 weeks post-ART initiation, most patients (83.5%, 81/97) achieved viral load suppression below 200 copies/mL. The log-rank test for time to viral load ≥200 copies/mL revealed significant inter-subtype differences (*P* = 0.0349), predominantly between CRF07_BC and BC subtypes (*P* = 0.0064), with CRF07_BC demonstrating faster suppression than BC (Figures 2A, B). By 24 weeks, 97.9% (95/97) of patients had suppressed viral load below 200 copies/mL. Survival analysis for viral load ≥200 copies/mL continued to show significant subtype-related differences (*P* = 0.0242), notably between CRF07_BC vs. BC (*P* = 0.0064) and CRF07_BC vs. the B+CRF01_AE/CRF55_01B group. CRF07_BC achieved suppression faster than both BC and B+CRF01_AE/CRF55_01B (Figures 2C–E). However, at subsequent follow-up time points (36 and 48 weeks), no significant differences in the survival curves for viral load ≥ 200 copies/mL were observed across subgroups (Figures 2F,G). At 36 weeks of ART, transient viral load rebound occurred in 2.1% (2/97) of participants. Both individuals were on the bictegravir/emtricitabine/tenofovir alafenamide (BIC/FTC/TAF) regimen without switching, and baseline transmitted drug resistance (TDR) testing revealed no resistance mutations in either case. At 48 weeks, 7.4% (6/81) of participants failed to suppress viral load below 200 copies/mL. Five were on BIC/FTC/TAF, and one on dolutegravir/lamivudine (DTG/3TC); none had switched regimens. Baseline TDR testing also showed no resistance mutations in these six participants. Subtype distribution of non-suppressors included BC (12.0%, 3/25), CRF01_AE (11.1%, 2/18), and CRF07_BC (3.1%, 1/32). Longitudinal virological outcomes corresponding to different antiretroviral regimens during the 48-week follow-up were analysed with stratification by HIV-1 subtype (Supplementary Table 3).

During the 48-week ART period, no significant differences were observed among participants of different subtypes in immunological parameters such as CD4+ T cell count, and the CD4+/CD8+ T cell ratio (Supplementary Table 1). The only significant difference between subtypes was found in triglyceride levels at week 24 of treatment (*P* = 0.041) (Supplementary Table 2). LOESS (locally weighted scatterplot smoothing) regression was used to model the trends in the changes of CD4+ T cell count, CD4+/CD8+ T cell ratio, and triglyceride levels (Supplementary Figure 7). Only the regression curves for CD4+ T cell count and triglycerides show some distinction between subtypes (Supplementary Figures 7A, C).

## 4 Discussion

HIV-1 genetic recombination and subtype diversification represent a global challenge for viral surveillance and antiretroviral intervention, particularly in regions with high epidemic activity (Alexiev and Dimitrova, 2025). In this study, we collected 105 cases of acute HIV-1 infection from a hospital in Shenzhen. Based on variations in the viral pol region sequences, we categorized the cases into CRF07_BC, other BC recombinants, CRF01_AE, B+CRF01_AE, CRF55_01B, B and C. This classification differs somewhat from the results of the HIV subtype epidemiological survey conducted by the Shenzhen Center for Disease Control and Prevention from 2011 to 2018. According to the 2011–2018 data, the predominant subtype in Shenzhen was CRF07_BC, followed by CRF01_AE, CRF55_01B, subtype B, CRF08_BC, unique recombinant forms (2.41%), and other 22 subtypes (1.99%) (Zhang D. et al., 2021). In our current HIV early infection cohort, we have observed a significant presence of other BC recombinant subtypes and a few B+CRF01_AE recombinant subtypes. Although some studies suggest that B+CRF01_AE recombinant strains may be associated with rapid disease progression and poor immune reconstitution (Ge et al., 2022), the current data indicate that timely initiation of ART during the early HIV infection phase does not affect immune recovery at 48 weeks of treatment. In addition to changes over time, the distribution of HIV subtypes in Shenzhen may differ, and there can also be variations in subtype distribution across other regions. The national HIV subtype distribution data from the China HIV Gene Sequence Data Platform differs from the subtype distribution observed in our early infection cohort. According to the 2018 national HIV subtype distribution data provided by the HIV molecular surveillance system on the platform, CRF01_AE accounted for 15.6%, CRF07_BC for 12.0%, subtype B for 10.6% (7958/75308), CRF08_BC for 2.7%, and other subtypes for 59.2% (HIV, 2024). These evolving subtype dynamics reflect the global trend of HIV-1 recombinant emergence, providing regional data that complements the global molecular epidemiological map of HIV-1.

MSM have become one of the high-risk groups for HIV infection in recent years (Schackman, 2021). In our study’s early infection cohort, most participants are MSM, with a small proportion being heterosexual. Studies have shown that rush poppers are widely used within the MSM population, and its use increases the risk of high-risk sexual behaviors, such as anal sex, group sex, and commercial sex work, thereby raising the likelihood of HIV and other sexually transmitted infections (Chen et al., 2018; Wang et al., 2020). CRF07_BC, a strain widely spread in China, is a recombinant variant of HIV-1 B′ and C found among injection drug users (PWID) in Southwest China during the 1990s (Ge et al., 2021b). It has since spread widely among both heterosexual and MSM populations (Ge et al., 2021b). Recently, the use of newer drugs like rush poppers may be contributing to the further spread of the CRF07_BC strain within the MSM community. The majority of MSM in our cohort are relatively young, but cases of early HIV infection in individuals over 50 years old have recently been observed. Our study revealed that only a subset of younger men who have sex with men (MSM) seek medical evaluation and obtain a diagnosis during the ultra-early phase of HIV-1 infection. Beyond promoting the benefits of early diagnosis and treatment among MSM, intensified medical outreach and educational initiatives targeting older populations are imperative to minimize transmission risks as effectively as possible. With China facing an aging population, the number of elderly individuals living with HIV is gradually increasing (Wang et al., 2023). Thus, it is essential to focus more on early education and prevention efforts within the elderly population.

Our study compared the clinical baseline and follow-up outcomes of individuals with different HIV subtypes, revealing that the B+CRF01_AE/CRF55_01B group is more likely to carry transmissible drug resistance mutations. The B+CRF01_AE/CRF55_01B group in this study includes the CRF01_AE/B and CRF55_01B subtypes, which exhibited common resistance mutations such as V179E and M184V. V179E is the primary resistance mutation in the CRF55_01B subtype, while M184V and V179E are the most prevalent resistance mutations in the CRF01_AE/B subtype. Our findings align with previous research on the drug resistance of CRF55_01B, which identified M184V (43.83%) as the most common mutation for NRTIs and V179E (98.77%) as the most prevalent mutation for NNRTIs (Lan et al., 2020; Zhang D. et al., 2021; Zhang et al., 2020). Currently, there are no studies specifically describing common transmissible drug resistance mutations for the CRF01_AE/B subtype. In our research, in addition to M184V and V179E, the CRF01_AE/B subtype also exhibited resistance mutations such as Y181C and E138G. Previous studies have also found potential genetic barriers to specific resistance mutations between HIV-1 subtypes (Mulu et al., 2015). The same resistance mutation sites can result in different drug sensitivities across various subtypes. For example, a study showed that integrase from subtype C, carrying the resistance mutations E92Q/N155H, was approximately 10 times more sensitive to each of the two integrase inhibitors (raltegravir and elvitegravir) compared to integrase from subtype B, which carried the same mutations (Bar-Magen et al., 2010). In our study cohort, we observed that CRF07_BC was potentially more sensitive to the current integrase inhibitor-based ART regimen during the early phase of treatment (24 weeks), compared to the BC recombinant and B+CRF01_AE recombinant subtypes. Although the commonly observed E157EQ mutation was found in the integrase region of CRF07_BC, the high resistance barrier of second-generation integrase inhibitors benefits treatment outcomes. Similar to etravirine (ETR) remaining effective in EFV treatment failure cases despite EFV-associated resistance mutations, it may also be useful for treating HIV-1 infections caused by different genetic variants, as HIV-1 gene variation does not significantly affect ETR sensitivity (Pereira-Vaz et al., 2012). Such subtype-specific TDR patterns are not unique to this cohort and may serve as a reference for identifying high-risk recombinant strains in other global regions with similar HIV-1 epidemic profiles.

Thyroid dysfunction is relatively common among patients with HIV infection. A multicenter cross-sectional study by Sonia Beltran et al. found that 16% of HIV-infected patients had hypothyroidism (Beltran et al., 2003). In our study, thyroid function impairment was not evident during the early stages of HIV infection. Although the median levels of T3 and FT3 in the CRF07_BC and BC groups were slightly lower than those in other groups, they remained within the normal range. Research by Mads Haslof et al. found no significant difference in the prevalence of overt hyperthyroidism or hypothyroidism between well-treated AIDS patients and HIV-negative subjects (Harsløf et al., 2018).

In our study, there were no significant differences in immune recovery, such as CD4+ T cell count and CD4+/CD8+ T cell ratio, among the different HIV subtypes in the early infection cohort after ART initiation, suggesting that early ART initiation does not lead to different long-term benefits for patients infected with different viral subtypes. Previous studies have shown that early ART initiation during the acute infection phase can limit the size of the viral reservoir, reduce symptoms caused by ARS, promote CD4+ T cell recovery, and improve endothelial dysfunction, resulting in long-term benefits for patients (Bush et al., 2019; Gabert et al., 2023; Lundgren et al., 2023; Sharma et al., 2019). In our research, no significant differences were observed in the recovery of immune cells in the short and long term among early-stage HIV-infected patients with different subtypes after ART. Only in the early stage of ART was it noticed that the CRF07_BC subtype seemed to be more sensitive in terms of viral load reduction compared to other subtypes, while no distinctions were found among various subtypes in the later stage. Currently, the treatment regimen mainly based on second-generation integrase inhibitors has enabled most patients to achieve viral suppression and immune recovery after 48 weeks, which is beneficial for the long-term benefits of early-stage HIV-infected patients. Other researchers have reported that the CRF01_AE/B recombinant strain in Nanjing was associated with faster disease progression and poorer immune recovery compared to CRF07_BC, with CD4^+^ T cell counts declining more rapidly and lower immune recovery potential, particularly in patients with baseline CD4^+^ T cell counts below 300 cells/μl before ART initiation (Ge et al., 2022). The researcher holds the view that immune recovery correlates with CD4^+^ T cell counts, rather than with different subtypes (Ge et al., 2022). Another study involving perinatally HIV-infected adolescents in Cameroon found no significant differences in immune recovery or viral suppression between the CRF02_AG and non-CRF02_AG groups following ART (Togna Pabo et al., 2023). The early viral suppression heterogeneity across subtypes highlights a virological feature that warrants global attention in the early management of HIV-1 infection, especially in areas dominated by recombinant strains.

This study has several limitations. One limitation of this study is that HIV-1 subtyping was solely performed based on the pol gene (PR-RT-IN), without parallel detection of gag and env genes (Hu et al., 2022). As reported in previous studies, gag gene sequencing helps refine subtype classification, and multi-gene or full-genome sequencing achieves higher accuracy for HIV-1 subtyping (Louwagie et al., 1993; Myers et al., 1992). We chose the pol gene for three practical reasons: it is a well-recognized reliable subtyping target, the assay conforms to WHO and national AIDS detection standards, and low viral loads among acute/early HIV-infected participants prevented universal full-genome sequencing (Li et al., 2026; Norris et al., 2006; Pineda-Peña et al., 2013). Further studies will utilize multi-gene combined analysis to optimize subtyping performance. In our study, we identified 8 patients who were treated with either BIC/FTC/TAF or DTG/3TC. Among them, 2 patients experienced a transient viral rebound at 36 weeks, and 6 patients had a viral rebound at 48 weeks. Notably, all these patients had no resistance mutations detected in their baseline transmission drug resistance testing. Cases of complete resistance to second-generation integrase inhibitors in clinical practice are rare. However, it remains crucial to closely monitor changes in plasma viral load and the viral reservoir in these patients. It is essential to collect plasma and PBMC samples from patients experiencing viral rebound, perform RNA and DNA sequencing, and assess the potential risk of developing resistance after long-term treatment. Restricted by questionnaire design, we failed to collect detailed sexual behavior covariates including partner quantity, unprotected intercourse frequency and partners’ HIV status, so we could not adjust confounding high-risk behaviors via multivariate regression, which may overestimate the association between rush popper use and CRF07_BC infection. Although we confirmed all popper use occurred before HIV seroconversion, we could not match drug exposure timing to exact high-risk contact events.

Collectively, these findings extend the global understanding of HIV-1 subtype-associated virological characteristics in early infection, offering evidence for optimizing molecular surveillance and precision treatment strategies worldwide.

## 5 Conclusion

Our research indicates that the B+CRF01_AE recombinant strain is more prone to transmitted drug resistance. The viral suppression of the BC recombinant strain and the B+CRF01_AE recombinant strain at 12 and 24 weeks after ART is inferior to that of the CRF07_BC subtype strain, which might be attributed to genetic variation and drug resistance. Therefore, it is of crucial importance to closely monitor viral load, drug resistance and viral reservoirs in clinical practice. A deeper understanding of the genetic variations of HIV strains is required.

## Data Availability

The original contributions presented in the study are publicly available. This data can be found here: https://www.ncbi.nlm.nih.gov/, accession numbers PZ576187–PZ576282.

## Footnotes

1
https://www.hiv.lanl.gov/content/sequence/QC/index.html

## References

[B1] Alexiev I. Dimitrova R. (2025). The origins and genetic diversity of HIV-1: Evolutionary insights and global health perspectives. *Int. J. Mol. Sci.* 26:10909. 10.3390/ijms262210909 41303393 PMC12653013

[B2] Bar-Magen T. Donahue D. A. McDonough E. I. Kuhl B. D. Faltenbacher V. H. Xu H. et al. (2010). HIV-1 subtype B and C integrase enzymes exhibit differential patterns of resistance to integrase inhibitors in biochemical assays. *AIDS* 24 2171–2179. 10.1097/QAD.0b013e32833cf265 20647908

[B3] Beltran S. Lescure F.-X. Desailloud R. Douadi Y. Smail A. El Esper I. et al. (2003). Increased prevalence of hypothyroidism among human immunodeficiency virus—infected patients: A need for screening. *Clin. Infect. Dis.* 37 579–583. 10.1086/376626 12905143

[B4] Bush K. N. V. Teel J. L. Watts J. A. Gore R. S. Alvarado G. Harper N. L. et al. (2019). Association of endothelial dysfunction and antiretroviral therapy in early HIV infection. *JAMA Netw. Open.* 2:e1913615. 10.1001/jamanetworkopen.2019.13615 31626317 PMC6813669

[B5] Chen H. Yang Y. Huang Y. Dai Y. Zhang J. (2018). Prevalence of poppers use and its sexual risks among men who have sex with men in Southwestern China: A cross-sectional study. *BMC Public Health* 18:1103. 10.1186/s12889-018-6010-8 30200922 PMC6131870

[B6] Chu Q. Zhang X. Lan J. Zhang Q. Wei T. Fu Y. et al. (2023). Prevalence and factors associated with late diagnosis among older adults living with HIV in liuzhou, China: 2010-2020. *J. Med. Virol.* 95:e28288. 10.1002/jmv.28288 36349389

[B7] Cowan E. A. McGowan J. P. Fine S. M. Vail R. Merrick S. T. Radix A. et al. (2021). *Diagnosis and Management of Acute HIV Infection, New York State Department of Health AIDS Institute Clinical Guidelines.* Baltimore, MD: Johns Hopkins University. 33074631

[B8] Crowell T. A. Colby D. J. Pinyakorn S. Fletcher J. L. K. Kroon E. Schuetz A. et al. (2018). Acute retroviral syndrome is associated with high viral burden, CD4 depletion, and immune activation in systemic and tissue compartments. *Clin. Infect. Dis.* 66 1540–1549. 10.1093/cid/cix1063 29228130 PMC5930255

[B9] Cui H. Geng W. Sun H. Han X. An M. Jiang Y. et al. (2019). Rapid CD4+ T-cell decline is associated with coreceptor switch among MSM primarily infected with HIV-1 CRF01_AE in Northeast China. *AIDS* 33 13–22. 10.1097/QAD.0000000000001981 30102662

[B10] De Clercq J. De Scheerder M.-A. Mortier V. Verhofstede C. Vandecasteele S. J. Allard S. D. et al. (2024). Longitudinal patterns of inflammatory mediators after acute HIV infection correlate to intact and total reservoir. *Front. Immunol.* 14:1337316. 10.3389/fimmu.2023.1337316 38250083 PMC10796502

[B11] Fiebig E. W. Wright D. J. Rawal B. D. Garrett P. E. Schumacher R. T. Peddada L. et al. (2003). Dynamics of HIV viremia and antibody seroconversion in plasma donors: Implications for diagnosis and staging of primary HIV infection. *AIDS* 17 1871–1879. 10.1097/00002030-200309050-00005 12960819

[B12] Gabert R. Lama J. R. Valdez R. Dasgupta S. Cabello R. Sanchez H. et al. (2023). Acute retroviral syndrome is associated with lower CD4 + T cell nadir and delayed viral suppression, which are blunted by immediate antiretroviral therapy initiation. *AIDS* 37 1103–1108. 10.1097/QAD.0000000000003511 36779502 PMC10355282

[B13] Ge Y. Liu Y. Fu G. Lu J. Li X. Du G. et al. (2022). The molecular epidemiological and immunological characteristics of HIV-1 CRF01_AE/B recombinants in nanjing. China. *Front. Microbiol.* 13:936502. 10.3389/fmicb.2022.936502 35910646 PMC9335199

[B14] Ge Z. Feng Y. Li K. Lv B. Zaongo S. D. Sun J. et al. (2021a). CRF01_AE and CRF01_AE cluster 4 are associated with poor immune recovery in Chinese patients under cART. *Clin. Infect. Dis.* 72 1799–1809. 10.1093/cid/ciaa380 32296820

[B15] Ge Z. Feng Y. Zhang H. Rashid A. Zaongo S. D. Li K. et al. (2021b). HIV-1 CRF07_BC transmission dynamics in China: Two decades of national molecular surveillance. *Emerg. Microb. Infect.* 10 1919–1930. 10.1080/22221751.2021.1978822 34498547 PMC8477959

[B16] Gulick R. M. Clifford Lane H. Agwu A. Arduino R. Badowski M. Baker J. (2024). *Guidelines for the Use of Antiretroviral Agents in Adults and Adolescents With HIV.* Bethesda, MD: DHHS Panel on Antiretroviral Guidelines. Available online at: https://clinicalinfo.hiv.gov/en/guidelines/hiv-clinical-guidelines-adult-and-adolescent-arv/whats-new

[B17] Han X. An M. Zhang W. Cai W. Chen X. Takebe Y. et al. (2013). Genome sequences of a novel HIV-1 circulating recombinant form, CRF55_01B, identified in China. *Genome Announc.* 1:e00050-12. 10.1128/genomeA.00050-12 23405298 PMC3569284

[B18] Harsløf M. Knudsen A. D. Benfield T. Nordestgaard B. G. Feldt-Rasmussen U. Nielsen S. D. (2018). No evidence of increased risk of thyroid dysfunction in well treated people living with HIV. *AIDS* 32 2195–2199. 10.1097/QAD.0000000000001954 30005023

[B19] Hassan A. S. Hare J. Gounder K. Nazziwa J. Karlson S. Olsson L. et al. (2021). A stronger innate immune response during hyperacute Human Immunodeficiency Virus Type 1 (HIV-1) infection is associated with acute retroviral syndrome. *Clin. Infect. Dis.* 73 832–841. 10.1093/cid/ciab139 33588436 PMC8423478

[B20] HIV (2024). *HIV Gene Sequences Database [WWW Document].* https://nmdc.cn/hiv/ (accessed 12.21.24).

[B21] Hollingsworth T. D. Anderson R. M. Fraser C. (2008). HIV-1 Transmission, by Stage of Infection. *J. Infect. Dis.* 198 687–693. 10.1086/590501 18662132

[B22] Hu X. Feng Y. Li K. Yu Y. Rashid A. Xing H. et al. (2022). Unique profile of predominant CCR5-tropic in CRF07_BC HIV-1 infections and discovery of an unusual CXCR4-tropic strain. *Front. Immunol.* 13:911806. 10.3389/fimmu.2022.911806 36211390 PMC9540210

[B23] Jiamsakul A. Chaiwarith R. Durier N. Sirivichayakul S. Kiertiburanakul S. Van Den Eede P. et al. (2016). Comparison of genotypic and virtual phenotypic drug resistance interpretations with laboratory-based phenotypes among CRF01_AE and subtype B HIV-infected individuals. *J. Med. Virol.* 88 234–243. 10.1002/jmv.24320 26147742 PMC4698354

[B24] Lan Y. Xin R. Cai W. Deng X. Li L. Li F. et al. (2020). Characteristics of drug resistance in HIV-1 CRF55_01B from ART-experienced patients in Guangdong, China. *J. Antimicrob. Chemotherapy* 75 1925–1931. 10.1093/jac/dkaa116 32300784

[B25] Leyre L. Kroon E. Vandergeeten C. Sacdalan C. Colby D. J. Buranapraditkun S. et al. (2020). Abundant HIV-infected cells in blood and tissues are rapidly cleared upon ART initiation during acute HIV infection. *Sci. Transl. Med.* 12:eaav3491. 10.1126/scitranslmed.aav3491 32132218 PMC7293182

[B26] Li J. Xu Y. Tang Y. Hao J. Song C. Mao W. et al. (2026). Evaluation of a HIV-1 drug resistance genotyping method based on high-throughput sequencing (HTS). *BMC Infect. Dis.* 26:838. 10.1186/s12879-026-12763-3 41851630 PMC13112854

[B27] Louwagie J. McCutchan F. E. Peeters M. Brennan T. P. Sanders-Buell E. Eddy G. A. et al. (1993). Phylogenetic analysis of gag genes from 70 international HIV-1 isolates provides evidence for multiple genotypes. *AIDS* 7 769–780. 10.1097/00002030-199306000-00003 8363755

[B28] Lundgren J. D. Babiker A. G. Sharma S. Grund B. Phillips A. N. Matthews G. et al. (2023). Long-Term benefits from early antiretroviral therapy initiation in HIV infection. *NEJM Evid.* 2:10.1056/evidoa2200302. 10.1056/evidoa2200302 37213438 PMC10194271

[B29] Marichannegowda M. H. Zemil M. Wieczorek L. Sanders-Buell E. Bose M. O’Sullivan A. M. et al. (2023). Tracking coreceptor switch of the transmitted/founder HIV-1 identifies co-evolution of HIV-1 antigenicity, coreceptor usage and CD4 subset targeting: The RV217 acute infection cohort study. *eBioMedicine* 98:104867. 10.1016/j.ebiom.2023.104867 37939456 PMC10665704

[B30] Markowitz M. Mohri H. Mehandru S. Shet A. Berry L. Kalyanaraman R. et al. (2005). Infection with multidrug resistant, dual-tropic HIV-1 and rapid progression to AIDS: A case report. *Lancet J.* 365 1031–1038. 10.1016/S0140-6736(05)71139-9 15781098

[B31] Mulu A. Maier M. Liebert U. G. (2015). Lack of integrase inhibitors associated resistance mutations among HIV-1C isolates. *J. Transl. Med.* 13:377. 10.1186/s12967-015-0734-3 26626277 PMC4665939

[B32] Myers G. MacInnes K. Korber B. (1992). The emergence of simian/human immunodeficiency viruses. *AIDS Res. Hum. Retroviruses* 8 373–386. 10.1089/aid.1992.8.373 1571197

[B33] Norris P. J. Pappalardo B. L. Custer B. Spotts G. Hecht F. M. Busch M. P. (2006). Elevations in IL-10, TNF-α, and IFN-γ from the earliest point of HIV Type 1 infection. *AIDS Res. Hum. Retroviruses* 22 757–762. 10.1089/aid.2006.22.757 16910831 PMC2431151

[B34] Pereira-Vaz J. Duque V. Pereira B. Mota V. Morais C. Saraiva-da-Cunha J. et al. (2012). Prevalence of etravirine resistance associated mutations in HIV-1 strains isolated from infected individuals failing efavirenz: Comparison between subtype B and non-B genetic variants. *J. Med. Virol.* 84 551–554. 10.1002/jmv.23232 22337292

[B35] Pineda-Peña A.-C. Faria N. R. Imbrechts S. Libin P. Abecasis A. B. Deforche K. et al. (2013). Automated subtyping of HIV-1 genetic sequences for clinical and surveillance purposes: Performance evaluation of the new REGA version 3 and seven other tools. *Infect. Genet. Evol.* 19 337–348. 10.1016/j.meegid.2013.04.032 23660484

[B36] Rockstroh J. Germany B. Ambrosioni J. Spain B. Molina J.-M. Guaraldi G. (2023). *European AIDS Clinical Society Guidelines 2023.* Brussels, Belgium: European AIDS Clinical Society. Available online at: https://www.eacsociety.org/guidelines/eacs-guidelines/

[B37] Schackman B. R. (2021). The value and challenge of frequent Human Immunodeficiency Virus (HIV) testing among young men who have sex with men in the United States. *Clin. Infect. Dis.* 73 e1936–e1937. 10.1093/cid/ciaa1060 32730624 PMC8492148

[B38] Sharma S. Schlusser K. E. de la Torre P. Tambussi G. Draenert R. Pinto A. N. et al. (2019). The benefit of immediate compared with deferred antiretroviral therapy on CD4+ cell count recovery in early HIV infection. *AIDS* 33 1335–1344. 10.1097/QAD.0000000000002219 31157663 PMC6561661

[B39] Sheehan D. M. Trepka M. J. Fennie K. P. Prado G. Madhivanan P. Dillon F. R. et al. (2017). Individual and neighborhood determinants of late HIV diagnosis among latinos, Florida, 2007–2011. *J. Immigrant Minority Health* 19 825–834. 10.1007/s10903-016-0422-2 27119364 PMC5083229

[B40] Togna Pabo W. L. R. Fokam J. Njume D. Takou D. Santoro M.-M. Nyasa R. B. et al. (2023). HIV-1 subtype diversity and immuno-virological outcomes among adolescents failing antiretroviral therapy in Cameroon: A cohort study. *PLoS One* 18:e0293326. 10.1371/journal.pone.0293326 37878637 PMC10599502

[B41] Wang D. Zhou M. Wang P. Zhang J. Mi Y. Cheng F. et al. (2023). Treatment-naïve people living with HIV aged 50 years or older in Beijing, China, 2010-2020: Joinpoint regression model analysis of surveillance data. *J. Int. AIDS Soc.* 26:e26193. 10.1002/jia2.26193 38054578 PMC10698805

[B42] Wang L. Santella A. J. Wei X. Zhuang G. Li H. Zhang H. et al. (2020). Prevalence and protective factors of HIV and syphilis infection among men who have sex with men in Northwest China. *J. Med. Virol.* 92 1141–1147. 10.1002/jmv.25622 31696951

[B43] Wilton J. Light L. Gardner S. Rachlis B. Conway T. Cooper C. et al. (2019). Late diagnosis, delayed presentation and late presentation among persons enrolled in a clinical HIV cohort in Ontario, Canada (1999–2013). *HIV Med.* 20 110–120. 10.1111/hiv.12686 30430742

[B44] Zhang D. Zheng C. Li Hanping Li Hao et al. (2021). Molecular surveillance of HIV-1 newly diagnosed infections in Shenzhen, China from 2011 to 2018. *J. Infect.* 83 76–83. 10.1016/j.jinf.2021.04.021 33932447

[B45] Zhang J. Huang X. Tang W. Chu Z. Hu Q. Liu J. et al. (2021). Rapid clinical progression and its correlates among acute HIV infected men who have sex with men in China: Findings from a 5-Year multicenter prospective cohort study. *Front. Immunol.* 12:712802. 10.3389/fimmu.2021.712802 34367176 PMC8339583

[B46] Zhang K. Wei B. Tang Z. Wei Y. Zhao Z. Li D. et al. (2020). Acute HIV infection in a large teaching hospital in western China: Clinical, virological, and molecular epidemiological characteristics. *J. Med. Virol.* 92 3288–3294. 10.1002/jmv.26282 32644261

