# Supplementary material for: Comparison of clinical characteristics in acute and early HIV-1 infection across different subtypes: a hospital-based cohort study
Source: Front Microbiol. 2026 Jul 17;17:1878265. doi: 10.3389/fmicb.2026.1878265 (PMC13423765; doi:10.3389/fmicb.2026.1878265)
Supplement: Supplementary file 1 [file Table_1.docx]

**Table S1** Follow-up changes in T-lymphocyte counts in acute and early HIV-1 infections of different subtypes after ART initiation (weeks 12–48)

| **T-lymphocyte counts {[IQR] values}** | **12w** | **24w** | **36w** | **48w** |
| --- | --- | --- | --- | --- |
| **CD4+ T cells (/µL)** |  |  |  |  |
| **P-value** | 0.167 | 0.619 | 0.219 | 0.706 |
| **CRF07_BC** | 551(112~908) | 540(147~1107) | 518(134~1197) | 565(199~1146) |
| **BC** | 509(315~1023) | 531(242~1220) | 585(322~832) | 571(277~1183) |
| **CRF01_AE** | 551(231~804) | 595(311~1079) | 665(355~973) | 624(258~1282) |
| **B+CRF01_AE/CRF55_01B** | 449(364~599) | 542(403~986) | 489(443~924) | 584(413~1098) |
| **CD8+ T cells (/µL)** |  |  |  |  |
| **P-value** | 0.443 | 0.703 | 0.141 | 0.767 |
| **CRF07_BC** | 960(500~2125) | 898(321~1773) | 782(287~2450) | 824(372~2287) |
| **BC** | 912(415~2819) | 853(376~2576) | 687(404~1824) | 790(403~1875) |
| **CRF01_AE** | 860(320~2084) | 896(516~2440) | 785(345~2010) | 821(245~2576) |
| **B+CRF01_AE/CRF55_01B** | 1064(451~1851) | 943(577~1179) | 1000(587~1643) | 821(712~1186) |
| **CD4+/CD8+ T cell ratio** |  |  |  |  |
| **P-value** | 0.618 | 0.553 | 0.131 | 0.564 |
| **CRF07_BC** | 0.56(0.13~1.24) | 0.59(0.15~1.22) | 0.63(0.21~1.43) | 0.67(0.22~1.56) |
| **BC** | 0.56(0.21~1.46) | 0.60(0.24~1.47) | 0.70(0.28~1.71) | 0.65(0.33~1.48) |
| **CRF01_AE** | 0.57(0.20~1.72) | 0.65(0.19~0.53) | 0.77(0.21~1.78) | 0.77(0.18~1.72) |
| **B+CRF01_AE/CRF55_01B** | 0.50(0.26~0.90) | 0.59(0.43~0.87) | 0.54(0.28~1.00) | 0.68(0.43~0.93) |

**Table S2** Follow-up changes in other laboratory parameters in acute and early HIV-1 infection subtypes following ART initiation (weeks 2–48)

| **Laboratory parameters median{[IQR] values}** | **2w** | **4w** | **8w** | **12w** | **24w** | **36w** | **48w** |
| --- | --- | --- | --- | --- | --- | --- | --- |
| **Neutrophils(10^3/µL)** |  |  |  |  |  |  |  |
| **P-value** | 0.854 | 0.709 | 0.997 | 0.358 | 0.345 | 0.259 | 0.334 |
| **CRF07_BC** | 3.00  (1.46~6.26) | 2.91  (1.36~5.90) | 2.78  (0.91~7.30) | 2.62  (1.36~5.97) | 3.04  (1.42~7.89) | 2.93  (1.02~7.27) | 3.03  (1.76~7.92) |
| **BC** | 3.00  (1.81~5.17) | 3.07  (1.43~4.41) | 2.66  (2.06~5.10) | 2.93  (1.86~4.73) | 3.03  (2.15~8.45) | 3.19  (2.31~5.00) | 2.92  (1.78~6.56) |
| **CRF01_AE** | 3.00  (1.80~5.90) | 2.84  (1.84~7.23) | 2.66  (1.57~5.41) | 2.57  (1.58~8.07) | 3.03  (1.80~6.30) | 3.13  (1.98~6.31) | 2.91  (1.51~4.68) |
| **B+CRF01_AE**  **/CRF55_01B** | 3.00  (1.73~3.88) | 2.92  (1.70~4.29) | 2.66  (1.70~4.29) | 2.81  (1.63~5.05) | 2.57  (1.84~4.55) | 2.91  (1.35~3.61) | 2.83  (1.35~3.92) |
| **Monocytes (10^3/µL)** |  |  |  |  |  |  |  |
| **P-value** | 0.957 | 0.471 | 0.718 | 0.821 | 0.851 | 0.940 | 0.640 |
| **CRF07_BC** | 0.50  (0.34~1.18) | 0.49  (0.30~0.81) | 0.46  (0.22~0.82) | 0.45  (0.23~1.00) | 0.48  (0.21~0.73) | 0.49  (0.19~0.81) | 0.46  (0.22~0.74) |
| **BC** | 0.50  (0.35~0.82) | 0.48  (0.33~0.99) | 0.49  (0.33~0.70) | 0.46  (0.29~0.85) | 0.48  (0.33~0.77) | 0.48  (0.29~0.90) | 0.49  (0.34~1.11) |
| **CRF01_AE** | 0.50  (0.24~1.10) | 0.47  (0.31~0.98) | 0.46  (0.33~0.97) | 0.47  (0.29~0.74) | 0.48  (0.25~0.78) | 0.48  (0.33~0.77) | 0.48  (0.25~1.08) |
| **B+CRF01_AE**  **/CRF55_01B** | 0.50  (0.37~0.73) | 0.48  (0.39~0.73) | 0.49  (0.38~0.65) | 0.51  (0.38~0.64) | 0.48  (0.39~0.65) | 0.48  (0.38~0.61) | 0.48  (0.39~0.56) |
| **Lymphocyte(10^3/µL)** |  |  |  |  |  |  |  |
| **P-value** | 0.679 | 0.724 | 0.576 | 0.516 | 0.276 | 0.330 | 0.902 |
| **CRF07_BC** | 2.09  (1.40~5.31) | 2.18  (1.45~4.05) | 2.10  (1.21~3.74) | 2.21  (1.45~3.56) | 2.22  (1.37~3.38) | 2.21  (0.80~4.15) | 2.13  (1.04~4.13) |
| **BC** | 2.09  (1.23~4.63) | 2.10  (1.20~3.75) | 2.15  (1.44~4.03) | 2.18  (1.13~4.30) | 2.13  (1.08~3.61) | 2.11  (1.23~3.55) | 2.15  (1.10~3.52) |
| **CRF01_AE** | 2.09  (1.11~3.41) | 2.16  (1.22~2.67) | 2.41  (1.41~3.23) | 2.01  (1.29~3.18) | 2.36  (1.16~2.72) | 2.37  (1.51~3.59) | 2.14  (1.14~3.71) |
| **B+CRF01_AE**  **/CRF55_01B** | 2.09  (1.25~5.51) | 2.38  (1.39~4.68) | 2.15  (1.53~3.45) | 2.42  (1.31~4.36) | 2.34  (1.31~2.89) | 2.21  (1.49~3.93) | 2.14  (1.64~2.94) |
| **Cholesterol(mmol/L)** |  |  |  |  |  |  |  |
| **P-value** | 0.670 | 0.756 | 0.977 | 0.819 | 0.380 | 0.582 | 0.203 |
| **CRF07_BC** | 4.31  (3.28~5.54) | 4.37  (2.72~6.57) | 4.56  (3.02~7.59) | 4.44  (2.84~7.86) | 4.38  (2.68~7.51) | 4.36  (2.67~9.82) | 4.28  (2.57~8.74) |
| **BC** | 4.31  (2.64~6.08) | 4.29  (2.72~7.30) | 4.56  (2.27~6.28) | 4.60  (2.67~8.03) | 4.59  (2.91~7.88) | 4.80  (2.94~7.23) | 4.73  (2.96~8.10) |
| **CRF01_AE** | 4.31  (3.18~6.30) | 4.32  (3.18~7.11) | 4.56  (3.15~5.89) | 4.55  (2.98~6.88) | 4.84  (3.41~6.25) | 4.71  (3.46~6.43) | 4.71  (3.60~6.46) |
| **B+CRF01_AE**  **/CRF55_01B** | 4.31  (3.35~5.73) | 4.10  (3.26~6.33) | 4.62  (3.06~5.88) | 4.26  (2.93~6.16) | 4.59  (3.60~5.89) | 4.86  (3.26~6.33) | 4.59  (3.24~6.08) |
| **Triglyceride(mmol/L)** |  |  |  |  |  |  |  |
| **P-value** | 0.266 | 0.757 | 0.276 | 0.306 | **0.041*** | 0.212 | 0.05 |
| **CRF07_BC** | 1.43  (0.73~7.84) | 1.47  (0.56~4.92) | 1.29  (0.49~3.66) | 1.27  (0.59~4.47) | 1.19  (0.39~3.17) | 1.24  (0.49~4.20) | 1.32  (0.61~3.72) |
| **BC** | 1.47  (0.54~4.49) | 1.39  (0.57~7.61 | 1.27  (0.37~4.77) | 1.51(0.55~10.65) | 1.40(0.49~13.57) | 1.57  (0.53~9.33) | 1.35  (0.50~9.96) |
| **CRF01_AE** | 1.43  (0.67~8.90) | 1.48  (0.56~6.38) | 1.42  (0.65~3.82) | 1.33(0.50~13.00) | 1.50  (0.72~4.15) | 1.32  (0.73~5.39) | 1.42(0.70~25.27) |
| **B+CRF01_AE**  **/CRF55_01B** | 1.17  (0.64~2.01) | 1.51  (0.91~2.46) | 0.96  (0.66~17.2) | 1.06  (0.65~4.83) | 0.89  (0.51~5.29) | 1.09  (0.48 ~1.92) | 1.00  (0.59~1.32) |
| **Creatinine(umol/L)** |  |  |  |  |  |  |  |
| **P-value** | 0.346 | 0.466 | 0.389 | 0.947 | 0.769 | 0.488 | 0.391 |
| **CRF07_BC** | 85  (63~135) | 87  (65~141) | 86  (61~137) | 90  (61~137) | 89  (60~127) | 89.5  (64~135) | 88.5  (67~134) |
| **BC** | 87.5  (73.7~130) | 86.5  (62 ~128) | 89.5  (57.2~143) | 89.5  (47 ~137) | 89  (60~123) | 91.5  (61~137) | 93.5  (64~124) |
| **CRF01_AE** | 85  (60.8~109) | 84  (64~107) | 86  (61~102) | 86  (69~105) | 89  (69~103) | 87  (74~109) | 87  (74~106) |
| **B+CRF01_AE**  **/CRF55_01B** | 84  (73.2~100) | 82.5  (71~100) | 80.5  (69~115) | 86  (73~114) | 87  (74~107) | 92.4  (78~122) | 91.5  (78~114) |
| **Glomerular**  **filtration rate(ml/min)** |  |  |  |  |  |  |  |
| **P-value** | 0.220 | 0.464 | 0.350 | 0.999 | 0.804 | 0.498 | 0.674 |
| **CRF07_BC** | 106.6(59.9~129.5) | 102.6(56.8~133.1) | 101.9(58.4~134.9) | 98.1(58.4~136.7) | 98.3(58.6~133.8) | 97.0(59.4~133.1) | 97.5(60.0~130.6) |
| **BC** | 106.6(55.2~121.8) | 100.9(56.3~132.0) | 102.6(48.9~128.3) | 100.6(51.5~141.8) | 98.9(58.6~133.8) | 97.3(51.5~132.9) | 91.3(58.0~127.1) |
| **CRF01_AE** | 106.6(60.6~123.2) | 103.3(62.0~126.7) | 102.6(67.3~120.7) | 101.8(65.9~123.8) | 98.9(64.5~120.5) | 99.7(70.3~122.1) | 96.6(72.2~118.5) |
| **B+CRF01_AE**  **/CRF55_01B** | 106.6(80.8~124.5) | 110.1(80.8~126.6) | 113.8(77.4~127.2) | 101.0(76.1~124.3) | 102.0(84.3~121.0) | 93.0(69.6~121.0) | 95.8(75.5~120.1) |

**Table S3** Longitudinal distribution of HIV-1 subtypes and virological outcomes stratified by initial antiretroviral regimens during 48-week ART follow-up (weeks 12–48)

| **Number of participants{n /virological failure n (%)}** | **12w** | **24w** | **36w** | **48w** |
| --- | --- | --- | --- | --- |
| **BIC/FTC/TAF** |  |  |  |  |
| **CRF07_BC** | 25/2 (8.0%) | 25/1 (4.0%) | 25/2 (8.0%) | 23/1 (4.3%) |
| **BC** | 24/5 (20.8%) | 24/0 (0.0%) | 24/0 (0.0%) | 22/3 (13.6%) |
| **CRF01_AE** | 18/2 (11.1%) | 18/0 (0.0%) | 18/0 (0.0%) | 14/1 (7.1%) |
| **B+CRF01_AE/CRF55_01B** | 7/1 (14.3%) | 7/0 (0.0%) | 7/0 (0.0%) | 4/0 (0.0%) |
| **Subtotal (BIC/FTC/TAF)** | 74/10 (13.5%) | 74/1 (1.4%) | 74/2 (2.7%) | 63/5 (7.9%) |
| **TDF/3TC/EFV** |  |  |  |  |
| **CRF07_BC** | 4/0 (0.0%) | 4/0 (0.0%) | 4/0 (0.0%) | 4/0 (0.0%) |
| **BC** | 3/2 (66.7%) | 3/0 (0.0%) | 3/0 (0.0%) | 2/0 (0.0%) |
| **CRF01_AE** | 2/1 (50.0%) | 2/0 (0.0%) | 2/0 (0.0%) | 1/0 (0.0%) |
| **B+CRF01_AE/CRF55_01B** | 1/0 (0.0%) | 1/0 (0.0%) | 1/0 (0.0%) | 1/0 (0.0%) |
| **Subtotal (TDF/3TC/EFV)** | 10/3 (33.3%) | 10/0 (0.0%) | 10/0 (0.0%) | 8/0 (0.0%) |
| **DTG/3TC** |  |  |  |  |
| **CRF07_BC** | 2/0 (0.0%) | 2/0 (0.0%) | 2/0 (0.0%) | 2/0 (0.0%) |
| **BC** | 2/0 (0.0%) | 2/0 (0.0%) | 2/0 (0.0%) | 1/0 (0.0%) |
| **CRF01_AE** | 2/0 (0.0%) | 2/1 (50.0%) | 2/0 (0.0%) | 2/1 (50.0%) |
| **B+CRF01_AE/CRF55_01B** | 0/0 (0.0%) | 0/0 (0.0%) | 0/0 (0.0%) | 0/0 (0.0%) |
| **Subtotal (DTG/3TC)** | 6/0 (0.0%) | 6/1 (16.7%) | 6/0 (0.0%) | 5/1 (20.0%) |
| **LPV/r/3TC/TDF** |  |  |  |  |
| **CRF07_BC** | 3/1 (33.3%) | 3/0 (0.0%) | 3/0 (0.0%) | 2/0 (0.0%) |
| **BC** | 1/1 (100.0%) | 1/0 (0.0%) | 1/0 (0.0%) | 1/0 (0.0%) |
| **CRF01_AE** | 1/0 (0.0%) | 1/0 (0.0%) | 1/0 (0.0%) | 1/0 (0.0%) |
| **B+CRF01_AE/CRF55_01B** | 1/0 (0.0%) | 1/0 (0.0%) | 1/0 (0.0%) | 0/0 (0.0%) |
| **Subtotal (LPV/r/3TC/TDF)** | 6/2 (33.3%) | 6/0 (0.0%) | 6/0 (0.0%) | 4/0 (0.0%) |
| **ANV/3TC/TDF** |  |  |  |  |
| **CRF07_BC** | 0/0 (0.0%) | 0/0 (0.0%) | 0/0 (0.0%) | 0/0 (0.0%) |
| **BC** | 0/0 (0.0%) | 0/0 (0.0%) | 0/0 (0.0%) | 0/0 (0.0%) |
| **CRF01_AE** | 0/0 (0.0%) | 0/0 (0.0%) | 0/0 (0.0%) | 0/0 (0.0%) |
| **B+CRF01_AE/CRF55_01B** | 1/1 (100.0%) | 1/0 (100.0%) | 1/0 (100.0%) | 0/0 (0.0%) |
| **Subtotal (ANV/3TC/TDF)** | 1/1 (100.0%) | 1/0(0.0%) | 1/0(0.0%) | 1/0 (0.0%) |
| **Overall Total** | 97/16 (16.5%) | 97/2 (2.1%) | 97/2 (2.1%) | 81/6 (7.4%) |

Column notation: {n/virological failure n (%)} = total number of participants under this regimen-subtype combination at the corresponding week / number of participants meeting virological failure criteria, (percentage of failure within the subgroup).


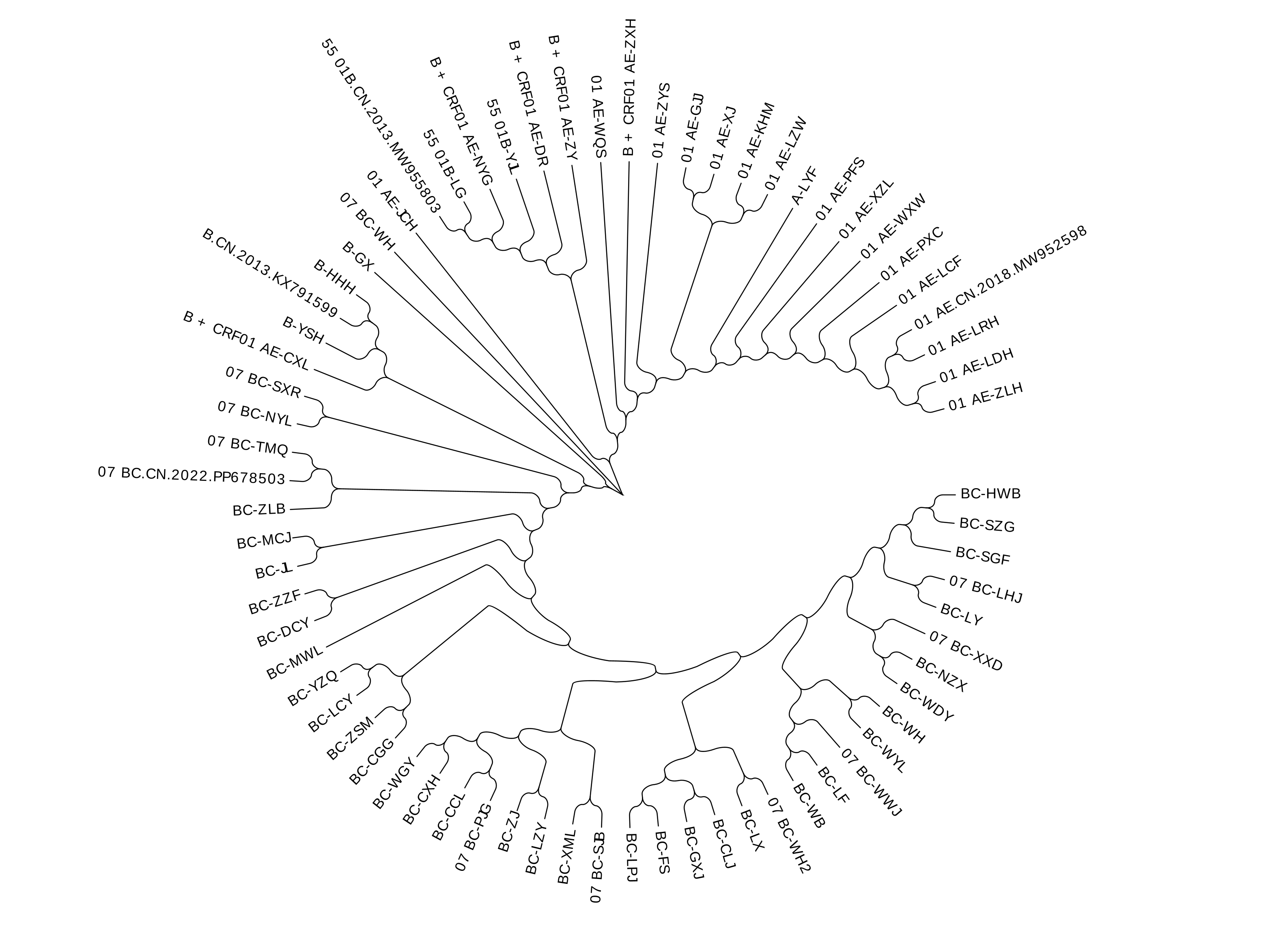


**Fig.S1** Phylogenetic tree of protease and reverse transcriptase gene sequences from a subset of acute and early HIV-1 infection cases.

**
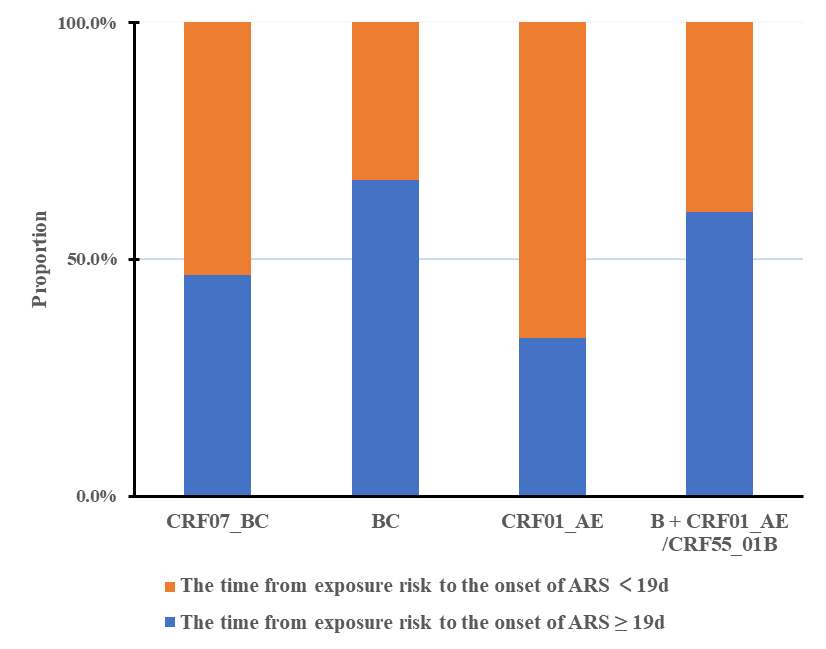
**

**Fig.S2** Proportional distribution of time intervals from suspected exposure to ARS onset among acute and early HIV-1 infection cases, stratified by subtype.

**
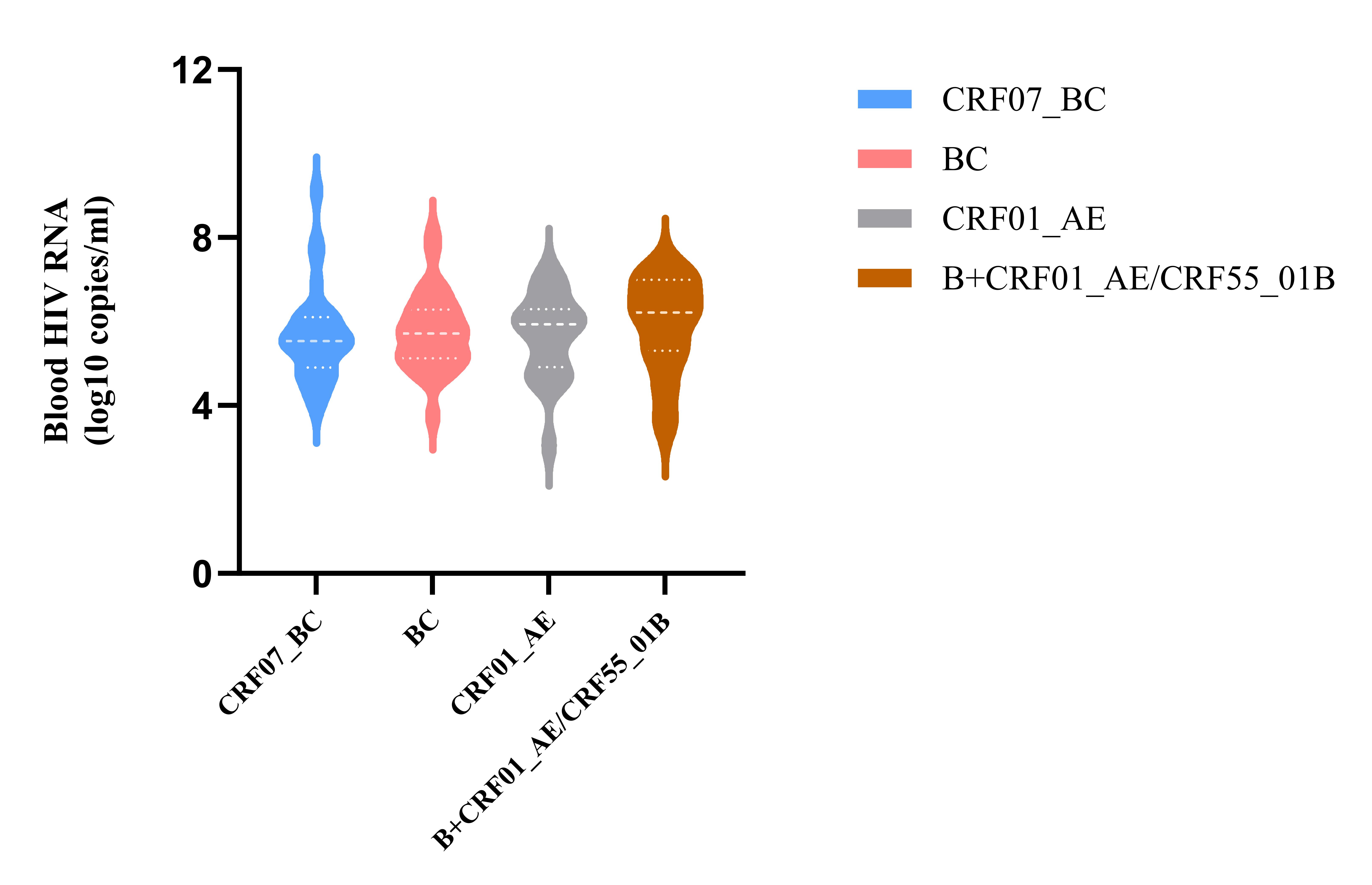
**

**Fig.S3** Plasma HIV-1 RNA levels in acute and early infection cases, stratified by subtype.


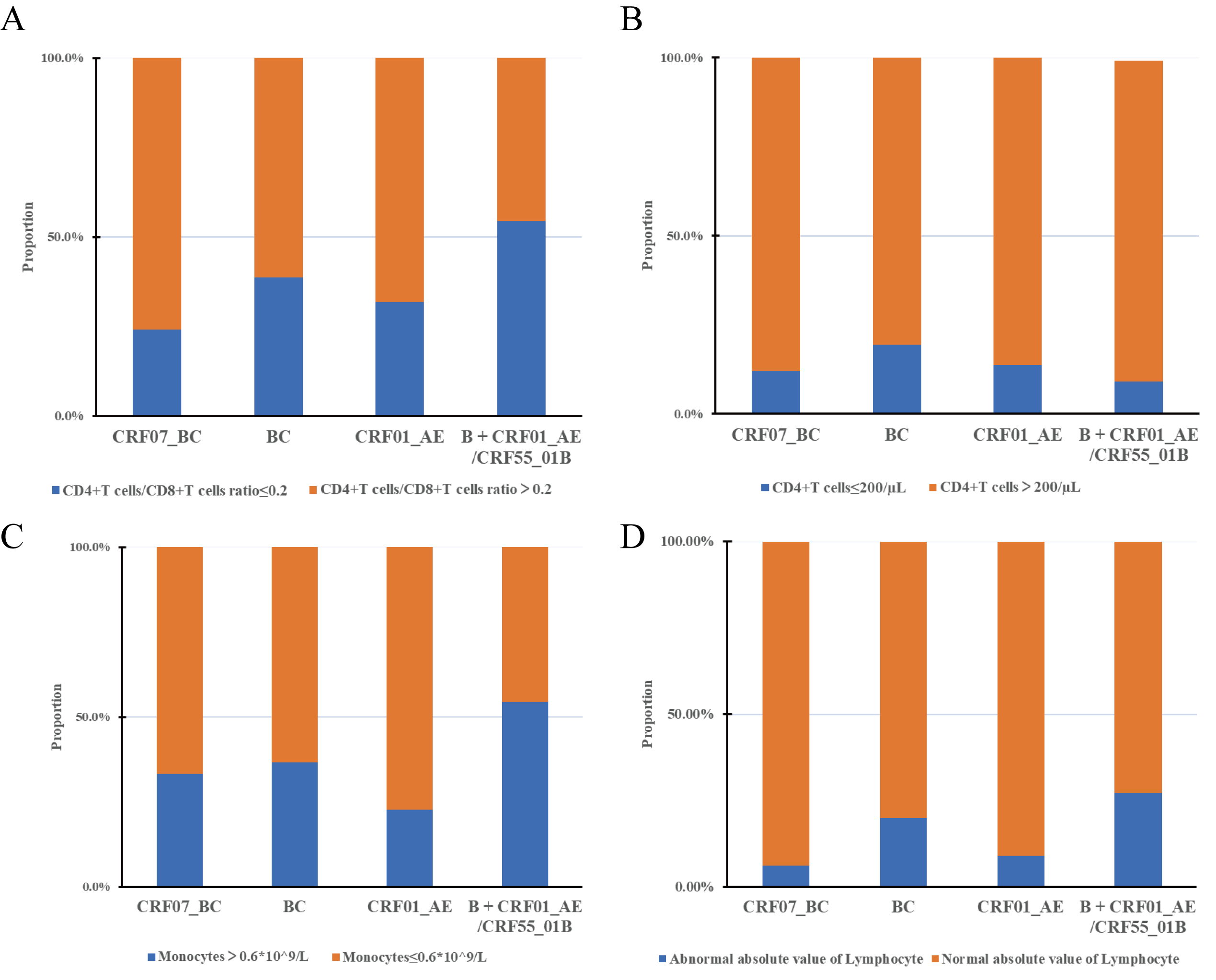


**Fig.S4** Baseline immunological characteristics of acute and early HIV-1 infection cases by subtype. (A) CD4⁺/CD8⁺ T-cell ratio distribution; (B) CD4⁺ T-cell count distribution; (C) monocyte count distribution; (D) lymphocyte count distribution.

**
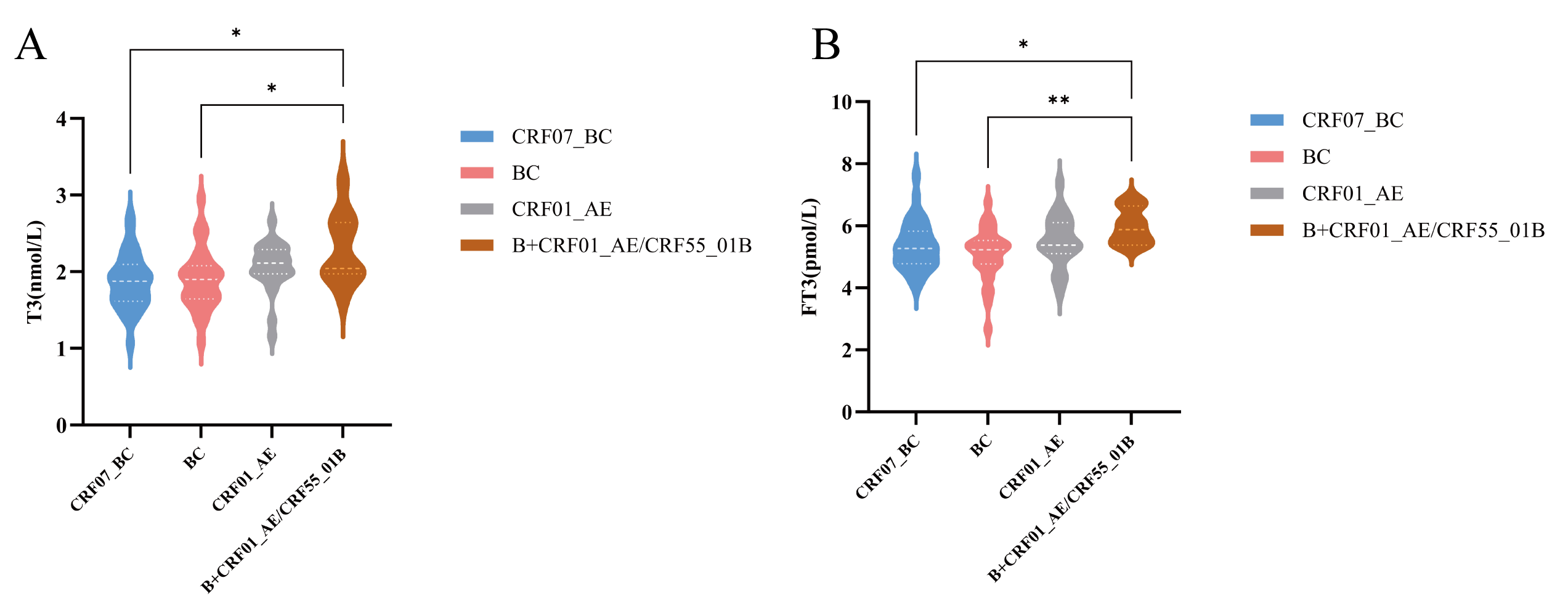
**

**Fig.S5** T3 and FT3 concentrations vary across viral subtypes during the acute and early HIV-1 infection phases. (A) Differences in T3 concentrations among acute and early HIV-1 infection subtypes. (B) Differences in FT3 concentrations among acute and early HIV-1 infection subtypes.

* P value < 0.05, ** P value < 0.01.

**
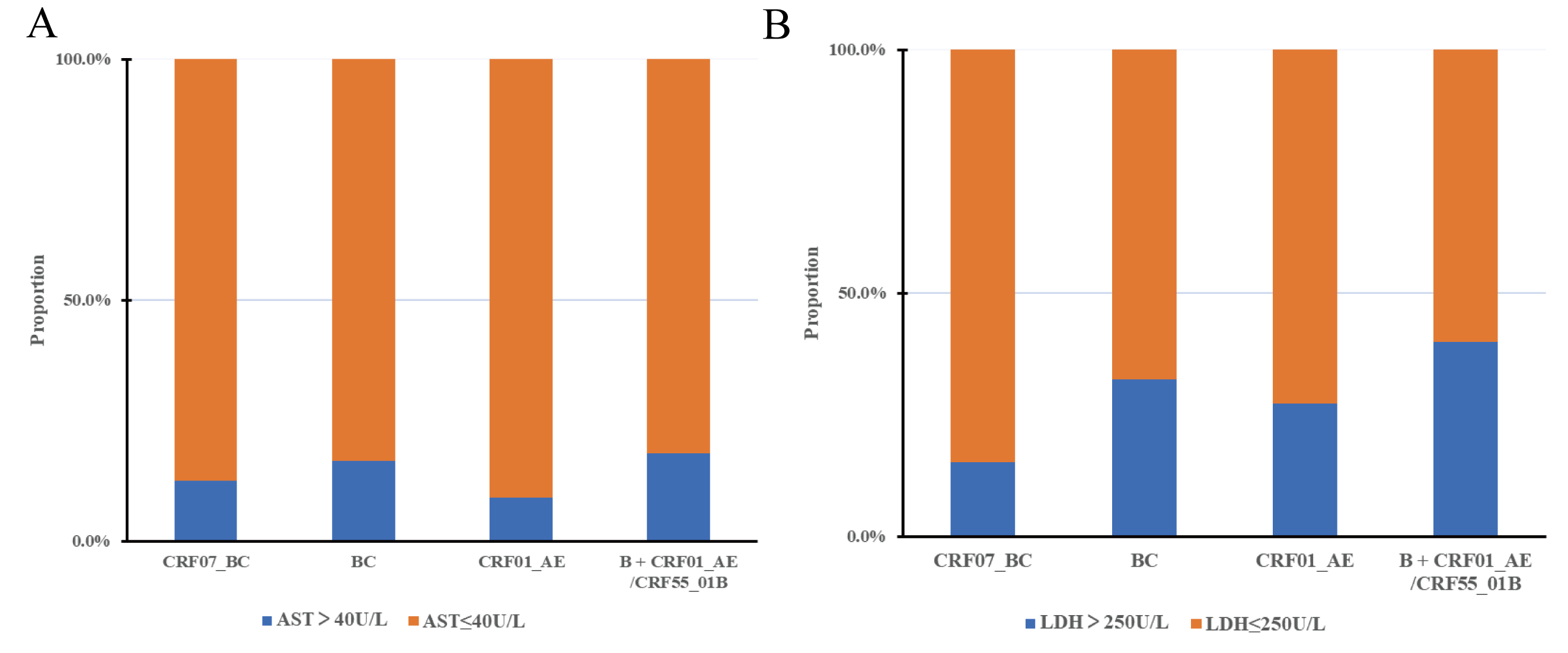
**

**Fig.S6** Comparison of abnormal AST and LDH proportions among acute and early HIV-1 infection subtypes. (A) Distribution of AST abnormalities; (B) Distribution of LDH abnormalities.


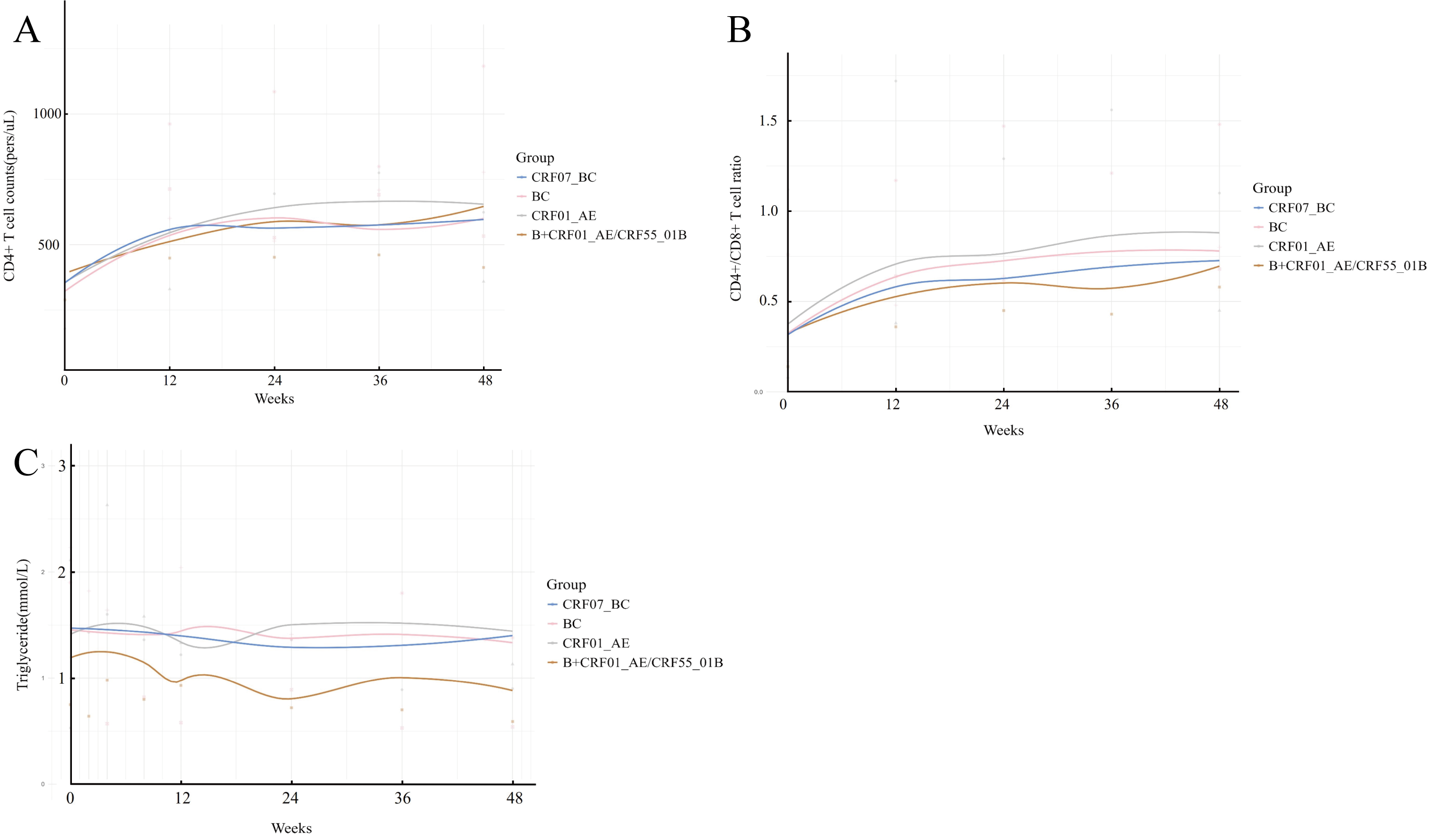


**Fig.S7** LOESS regression curves depicting 48-week post-ART longitudinal changes in T-lymphocytes and selected laboratory parameters among acute and early HIV-1 infection cases across different subtypes. (A) CD4⁺ T-cell count trajectories; (B) CD4⁺/CD8⁺ T-cell ratio dynamics;(C) Triglyceride level trends.
